# Mucin-derived sugars act as metabolic brakes controlling growth initiation in *Akkermansia muciniphila*

**DOI:** 10.1080/19490976.2026.2691334

**Published:** 2026-06-26

**Authors:** Bryan D. Lakey, Katherine J. Wozniak, Robert A. Britton, Jeffrey J. Tabor

**Affiliations:** a Department of Bioengineering, Rice University, Houston, TX, USA; b Department of Molecular Virology & Microbiology, Baylor College of Medicine, Houston, TX, USA; c Alkek Center for Metagenomics and Microbiome Research, Baylor College of Medicine, Houston, TX, USA; d Department of Biosciences, Rice University, Houston, TX, USA; e Department of Chemical and Biomolecular Engineering, Rice University, Houston, TX, USA; f Rice Synthetic Biology Institute, Rice University, Houston, TX, USA

**Keywords:** *Akkermansia muciniphila*, microbial metabolism, central carbon metabolism, lag phase, mucin, glycans, gut colonization

## Abstract

*Akkermansia muciniphila* is a key member of the gut microbiota and plays important roles in host metabolism and health. In the colon, *A. muciniphila* extracts nutrients from oligosaccharide-rich mucin glycans that comprise the mucosa. However, this environment is complex and shaped by dietary inputs, microbiome metabolism, and mucin glycan composition varying across hosts, gastrointestinal regions, and physiological states. How strains of *A. muciniphila* integrate these nutrient signals into growth initiation and niche colonization remains unclear. Here, we compare physiological responses of a human- and mouse-derived strain of *A. muciniphila*, finding that dietary sugars differentially affect these isolates, suggesting host-associated tuning of metabolic capacity. In contrast, several mucin-derived sugars impose a conserved, concentration-dependent delay in growth initiation, implicating the lag phase as a critical metabolic checkpoint for growth. Genetic suppressor analysis identified sugar kinases and a component of the tricarboxylic acid cycle as genetically encoded control points linking glycan sugar exposure to the energy balance required for growth. These findings demonstrate that mucin-derived sugars function as both nutrients and metabolic stressors, regulating growth initiation. We propose that *A. muciniphila* employs metabolic “brakes” to coordinate growth with mucin composition, putatively linking host glycan landscapes to microbial physiology and ecological fitness within the mucus layer.

## Introduction

The colonic epithelium is covered by a dense and stratified mucus matrix. While the inner layer of this matrix is tightly packed and functions as a physical barrier separating the microbiota from host cells, its continuous secretion and expansion towards the lumen creates a mesh-like outer region that serves as both a nutrient reservoir and an anchoring platform.^1-3^ This mesh-like region mediates host‒microbe symbiosis and metabolic exchange.^4^ Here, specialized mucolytic bacteria work in concert to enzymatically cleave and extract nutrients from oligosaccharide-rich mucin glycoproteins that comprise this matrix. This process liberates soluble mono- and oligosaccharides that diffuse into this niche and support the growth of non-mucolytic commensals.^5^ ^,^ ^6^ As most simple dietary sugars are absorbed in the small intestine,^7^ these host-derived glycans provide an abundant and stable nutrient source for gut bacteria. In return, bacterial degradation and fermentation of these accessible glycans supply host epithelia with beneficial short-chain fatty acids,^8^ help maintain the resilience of the inner barrier by stimulating the secretion of nascent mucin,^9^ and promote microbial diversity by releasing simpler sugars for non-mucolytic commensals.^5^ ^,^ ^6^

MUC2 is the dominant secreted mucin in the colon.^1^ This protein is heavily *O*-glycosylated by core structures of *N*-acetylglucosamine (GlcNAc), *N*-acetylgalactosamine (GalNAc), and d-galactose (d-gal) and is decorated with terminal l-fucose (l-fuc) and *N*-acetylneuraminic acid (Neu5Ac/sialic acid) residues. This glycan architecture generates diverse sugar configurations that vary with tissue type along the gastrointestinal (GI) tract.^10^ ^,^ ^11^ For example, fucosylation decreases while sialylation increases longitudinally toward the distal colon in humans.^10^ ^,^ ^12^ These changing glycosylation patterns shape the ecological and metabolic landscape for mucolytic and other commensal bacteria.^6^

The Gram-negative anaerobe *Akkermansia muciniphila* is a cornerstone member of the gut microbiota and a model mucolytic bacterium in the lower GI tract of humans^13^ and other animals.^14^ *A. muciniphila* encodes a large repertoire of glycosidases and accessory enzymes that degrade mucin *O*-glycans, enabling it to utilize GlcNAc and/or GalNAc as sole carbon and nitrogen sources.^15^ Elevated *A. muciniphila* abundance and the corresponding synthesis of lipids and short-chain fatty acids have been linked to improved GI barrier integrity^16^ ^,^ ^17^ and beneficial immunomodulation.^18^ More broadly, *A. muciniphila* is thought to play key roles in host GI and metabolic health, and its abundance is inversely correlated with obesity,^19^ hyperglycemia,^20^ Crohn’s disease,^21^ and ulcerative colitis.^22^

Previous studies of *A. muciniphila* have primarily focused on mucin-degradation pathways and subsequent metabolism of mucin-derived monosaccharides, providing valuable insight into broad glycan substrate utilization and enzymatic function.^23-29^ Furthermore, single-carbon media formulations have been used to identify which mucin-derived monosaccharides can support growth.^23-25^ However, the use of such media to study bacterial metabolism often fails to capture context-dependent metabolic interactions such as stress, nutrient competition, or cooperative carbon use that can emerge in mixed or energy-limited environments. Consequently, the broader carbon utilization capacity of *A. muciniphila*, as well as the metabolic constraints and stress responses that shape cell physiology, remain poorly understood.

Strain-level variation in *A. muciniphila*, including metabolism, also remains understudied. Anatomical and dietary differences between humans and mice, including colonic mucin composition,^30^ likely drive bacterial strain divergence that may influence probiotic efficacy and preclinical development of this bacterium as a therapeutic.^31^ The strain *A. muciniphila* JAX-AM001 originated from a C57BL/6 laboratory mouse maintained on a high-fiber standardized chow diet,^32^ whereas the well-studied type strain BAA-835 was isolated from a fecal sample of a human with a more diverse diet.^13^ Although both strains have been studied, systematic comparisons of their metabolic responses under defined conditions have not been performed. Comparing human- and mouse-derived *A. muciniphila* isolates would provide an opportunity to distinguish conserved physiological mechanisms from host-adaptive traits, possibly informing how diet and mucosal glycan composition shape strain-specific metabolic responses and beneficial functions.

Here, we utilize a defined growth medium to compare the growth profiles of these two *A. muciniphila* strains. We found that the growth of human-derived BAA-835 is often enhanced by common dietary sugars, while mouse-derived JAX-AM001 is frequently inhibited by them. Despite these differences, both isolates are sensitive to several mucin-derived monosaccharides, including d-gal, l-fuc, and Neu5Ac, with pronounced growth inhibition during the lag phase. This effect is strongest in JAX-AM001, revealing strain-specific tuning that governs vulnerability during the transition from dormancy to active growth. Leveraging the heightened sensitivity of JAX-AM001, we isolated spontaneous suppressor mutants that restore growth under inhibitory conditions. Genomic analysis of these mutants identifies lesions in sugar kinases and a component of the tricarboxylic acid (TCA) cycle, implicating carbon overflow and energy imbalance as central constraints on early growth. Together, these findings reveal that mucin-derived sugars can function as both nutrients and stressors, transiently disrupting metabolic balance at a critical physiological checkpoint. More broadly, our results provide a framework for understanding how host glycan composition and quality shape the ecological fitness of *A. muciniphila* within the mucus layer.

## Materials and methods

### Culturing of *Akkermansia muciniphila*

Two strains of *A. muciniphila* were used in this study: ATCC BAA-835 (Muc^T^ [CIP 107961] isolated from human feces) and MDA JAX-AM001 (murine fecal isolate). Both strains were grown in *Akkermansia* defined media (ADM), modified from.^13^ ^,^ ^18^ ^,^ ^33^ ADM contains 50 mM MOPS, 4 mM tricine, 25 mM NaCl, 20 mM NaHCO_3_, 2.2 mM KH_2_PO_4_, 1.8 mM K_2_HPO_4_, 5.6 mM NH_4_Cl, 50 mM L-threonine, 25 mM N-acetylglucosamine, and 5.7 mM L-cysteine, as well as 0.5 mM K_2_SO_4_, 1 mM MgCl_2_, and 1 mM CaCl_2_ added from 1000x liquid stocks. The pH was adjusted to 7 using KOH and filter-sterilized. Immediately before use 100 µL FeSO_4_ (5  mM stock, filter-sterilized and acidified; final 10  µM), 10 µL trace vitamins (5x stock (g/50 mL): 0.005 g biotin, 0.05 g niacin, 0.125 g pyridoxine, 0.025 g riboflavin, 0.05 g thiamine, 0.025 g cyanocobalamine, 0.025 g *p*-aminobenzoic acid, 0.025 g pantothenic acid), and 500 µL ATCC trace mineral supplement (ATCC MD-TMS) were added per 50  mL of ADM. For agar plates, 1.5% agar was added to the base ADM media and autoclaved. Once cooled, FeSO_4_, vitamin and mineral reagents were added. When required, the media was supplemented with 10  µg/mL gentamicin,^33^ or 0.4% (w/v) porcine gastric mucus (Type II mucin from Porcine stomach, Sigma–Aldrich). Plates and liquid cultures were grown either in anaerobic jars (Almore International) compatible with GasPack EZ Gas Serachels (Fisher Scientific) or under an anaerobic atmosphere (5% CO_2_, 5% H_2_, 90% N_2_) inside a vinyl anaerobic chamber (COY Laboratory Products) and incubated at 37 °C.

### Growth analysis

Single colonies from a fresh plate were used to inoculate separate 5 mL cultures of ADM media and grown anaerobically for ~30 h until the cells reached mid-log phase growth. Each culture was then back-diluted and normalized to OD_600_ = 0.2. Then, 10 µL of each culture was seeded into 200 µL of reduced media in a clear 96-well plate (final OD_600_ 0.01). The plates were sealed with clear sealing film and incubated in a microplate reader (Sunrise by Tecan Life Sciences) within the anaerobic chamber for 60 h. The OD_600_ was measured every 30 min after 5 s of orbital shaking. The growth profiles of each isolate and sugar were measured in triplicate. The doubling time was calculated by determining the linear range of growth fit to an exponential growth model: N_t_ = N_0_e^rt^, approximating the doubling time as ln(2)/r (N_t_ = final OD_600_; N_0_ = initial OD_600_; r = growth rate (doubling time, T_d_); t = time (h) between N_t_ and N_0_ measurements). Unless noted, all sugars were added from a 50x stock to a final concentration of 20  mM.

For Hungate tube experiments, 14 mL of reduced ADM was inoculated to an OD_600_ of 0.05 from a fresh overnight culture. The cultures were then allowed to reach early log-phase at ~0.4 OD_600_ (~18 h) before being sealed with self-sealing rubber septa and open-top caps. The tubes were then removed from the anaerobic chamber, and the rubber septa were bleached to prevent contamination before the appropriate sugar was added using a sterile needle syringe (final concentration of 20 mM). Each culture was then grown at 37 °C, and OD_600_ was directly monitored using a spectrophotometer.

### Mutant isolation

As above, an overnight culture grown in ADM was diluted 1:100 into fresh ADM supplemented with permissive amounts of each sugar that severely delayed but did not prevent growth (d-ara = 0.1  mM; d-gal = 5  mM; l-fuc = 20  mM; determined from Figures S3 and S6). Cultures were grown as described above until dense (OD_600_ ~ 1.0) and then back-diluted 1:100 twice more in fresh media containing the same amount of each sugar. After the third and final passage reached OD_600_ = 0.5, 200 µL was spread onto ADM agar containing d-ara = 20 mM, d-gal = 20 mM, or l-fuc = 40 mM, respectively. The plates were incubated as described above until colonies formed. Individual colonies were streaked for isolation on fresh ADM agar plates containing the above sugars. From these restreaks, isolated colonies were picked, grown overnight in ADM containing the appropriate sugar, and freezer stocks of each culture were made (stored in 20% glycerol).

### Genomic DNA extraction

High-molecular weight DNA was extracted from strains of *Akkermansia muciniphila* using silica-column purification adapted from.^34^ A total of 10  mL of culture grown overnight (OD_600_ > 0.5) was harvested by centrifugation at 10,000 × *g* for 10  min at room temperature. The supernatant was carefully removed with a pipette. Each pellet was then resuspended in 200 µL of lysis buffer (50 mM Tris-HCl, pH 8, 10 mM EDTA, pH 8, and 1% Triton X-100). RNase A was then added to a final concentration of 0.5  mg/mL, and incubated at 37 °C for 30  min. Following incubation, 30 µL 10% SDS and 40 µL 10 mg/mL proteinase K were added, mixed well by gently pipetting with a wide-bore tip, and incubated at 55 °C for 30 min. Following incubation, ~30 µL of 3 M NaOAc (pH 5.2) and 600 µL of PB buffer (Qiagen) were added and mixed well. The entire mixture was then loaded onto a standard silica spin column and centrifuged at 15,000 × *g* for 1 min. The flow-through was discarded, and 500 µL of PB (Qiagen) was loaded onto the column, centrifuged as before, and the flow-through was discarded. The silica membrane was then washed with 750 µL of PE buffer (Qiagen), centrifuged, flow-through discarded, and then recentrifuged to evaporate any residual ethanol. The genomic DNA bound to the silica membrane was then eluted twice with 50 µL aliquots of nuclease-free water preheated to 65 °C.

### Sequencing and SNP analysis

The genomes were sequenced using Oxford Nanopore long reads performed by Plasmidsaurus at ~100x coverage per genome. SNP calling was performed using Breseq version 0.39.0 with all default settings.^35^ Strain-specific mutations were mapped to the respective genomes of MDA JAX-AM001 (Genome assembly ASM2681010v1; NCBI GenBank assembly: GCA_026810105.1) and ATCC BAA-835 (Genome assembly ASM2022v1; NCBI GenBank assembly: GCA_ 000020225.1). Individual SNPs were confirmed by colony PCR using primers flanking the locus of interest (Supplemental Data 4). The PCR fragments were amplified using Q5 Polymerase (New England Biolabs), purified (Qiagen PCR clean-up), and amplicon sequencing was performed by Plasmidsaurus.

### Protein modeling

Protein structure models for Amuc_1591, GalK (Amuc_0969), SucC (Amuc_1713), and FclK (Amuc_1830) were generated using ColabFold v1.5.5.^36^ For each protein, we ran the monomer workflow with default parameters and multiple sequence alignments built by MMseqs2 against the ColabFold sequence and environmental databases. The top-ranked model for each target, selected by pLDDT, was subjected to AMBER relaxation and used for all structural analyses. Protein sequences were taken from the corresponding genome annotations and are provided in Supplemental Data 5.

### Genomic comparison with BRIG

Comparative genome analysis of *Akkermansia muciniphila* BAA-835 and JAX-AM001 was performed using the BLAST Ring Image Generator (BRIG v0.95).^37^ The complete genome of BAA-835 (GenBank accession GCF_000020225.1) and the genome of JAX-AM001 (BV-BRC Genome ID 239935.3058) were each used as references in separate analyses to ensure symmetric comparisons. For each reference genome, the other strain’s assembly was formatted as a BLAST database and aligned using BRIG default settings, which apply BLASTn across sliding windows with percent-identity thresholds of 50%, 70%, and 100%. Circular plots were generated to visualize genome-wide nucleotide identity, GC content, GC skew, and to highlight regions of divergence. Annotations for both genomes were obtained from their respective GenBank files and used to label loci uniquely present, absent, or diverged between strains.

## Results

### Dietary sugars alter *A. muciniphila* growth in a strain-specific manner

**Figure 1.**
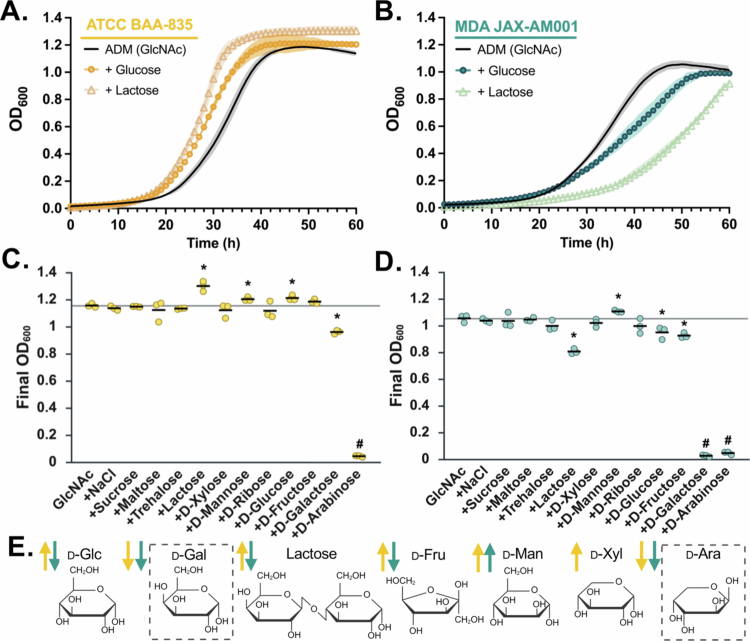
Strain-specific effects of common dietary sugars on the growth of *A. muciniphila*. (A, B) Growth data for *A. muciniphila* strain ATCC BAA-835 (human isolate, yellow) and MDA JAX-AM001 (murine isolate, teal) were measured in triplicate (three separate biological replicates across different days). The cells were cultured in *Akkermansia* defined media (ADM) and supplemented with 20  mM sugar. (C, D) Final cell density (OD_600_) of strain BAA-835 (yellow) and JAX-AM001 (teal) cultured in ADM supplemented with common mono- and disaccharides (+20 mM). Final OD_600_ of three replicates and the mean value (black bar) compared to the media-alone control (gray line). *p-value < 0.05; ^#^ *p*-value < 0.001. Final OD_600_ measurements from the growth curves are shown in Figures S1 and S2. (E) Structures of sugars were observed to have a positive or negative effect on the growth of each strain (arrows). d-gal and d-ara (boxes) both severely inhibited the growth of both strains.

To compare the sugar-dependent growth profiles of BAA-835 and JAX-AM001, we developed a modified GlcNAc medium that can be supplemented with different carbon sources (*Akkermansia* Defined Medium, ADM). Consistent with previous reports,^23^ ^,^ ^24^ we found that BAA-835 grows slightly faster in ADM supplemented with 20  mM d-glucose (doubling time (T_d_) = 4.0 h) or lactose (T_d_ = 3.9 h) than in ADM alone (T_d_ = 4.6 h) (Figure 1A, Table S1). Cultures grown with either sugar also appear to enter the exponential phase sooner than in ADM alone, consistent with improved growth (Figure 1A). Unexpectedly, we found that JAX-AM001 grows far more slowly in ADM supplemented with either d-glucose or lactose (T_d_ = 7.8 h or 8.9 h, respectively) than without (T_d_ = 5.4 h) (Figure 1B, Table S1). When grown in ADM supplemented with lactose, JAX-AM001 also exhibited an extended lag phase (~20 h) compared to ADM alone (~12 h) (Figure 1B). The contrasting D-glucose- and lactose-dependent growth profiles of BAA-835 and JAX-AM001 prompted us to perform a broader characterization of the impact of sugars on the growth of these two strains.

To this end, we repeated these growth experiments with a panel of dietary mono- and disaccharides and sugar alcohols (20 mM each). Similar to D-glucose and lactose, we found that BAA-835 doubles faster in the presence of d-fructose, d-xylose, and d-mannose (Figure S1, Table S1), with the latter also supporting a higher final cell density (Figure 1C). On the other hand, JAX-AM001 also grows more slowly in the presence of d-fructose and arabitol, suggesting that the growth of this strain is broadly inhibited by sugars and sugar alcohols. Interestingly, we found that d-mannose has a growth-stimulating effect on JAX-AM001 (Figure S2, Table S1). d-mannose, while commonly found in fruits, is also a component of transmembrane *N*-linked glycans found on the apical surface of colonic epithelial cells and as a minor component of MUC2 mucins.^2^ ^,^ ^3^ The growth-stimulating effects of this sugar on both strains suggest that *A. muciniphila* has a conserved capacity to metabolize sugars present in diverse host glycan structures.^38^ Finally, we observed that d-galactose (d-gal) imposes a partial growth defect on BAA-835 while d-arabinose (d-ara) results in complete growth inhibition (Figure 1C, Figure S1). For JAX-AM001, both sugars completely inhibited growth (Figure 1D, Figure S2). The shared sensitivity of these two strains to d-gal and d-ara suggested that *A. muciniphila* has conserved sugar-specific stress mechanism(s) (Figure 1E). We next used a genetics-based approach to explore these mechanisms.

### Stereospecific growth inhibition of *A. muciniphila* by d-arabinose at micromolar concentrations

**Figure 2.**
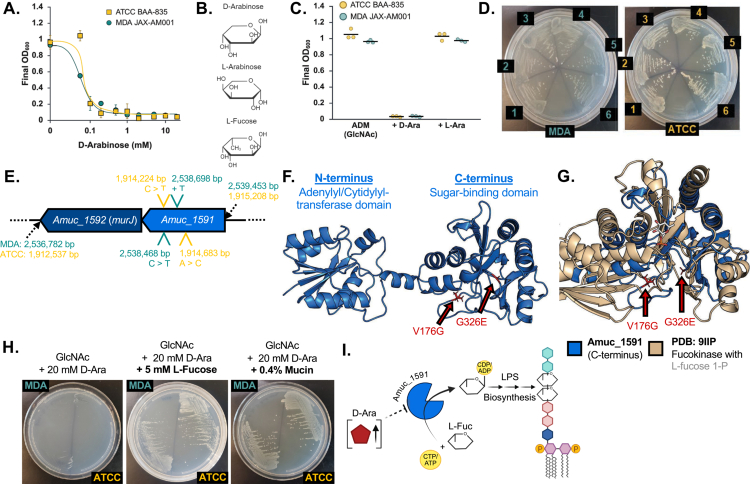
The stereospecific effects of d-ara on *A. muciniphila* growth are rescued by inactivation of a putative fucose-nucleotidyltransferase. (A) Dose‒response curves for the growth (final OD_600_) of BAA-835 and JAX-AM001 supplemented with d-ara. (*n* = 3; biological replicated measured on different days). (B) Structural configurations of d- and l-ara compared to l-fuc. (C) Final cell densities (OD_600_) of strains BAA-835 and JAX-AM001 grown in media supplemented with d- or l-arabinose, measured in biological triplicate. (D) Growth of isolated mutants (1–6) relative to the parental strain (WT), on agar plates containing 20 mM d-ara. (E) The genomic region, predicted transcriptional unit, and the position of each mutation identified within *Amuc*_*1591*. (F) Protein structure model of Amuc_1591 built using ColabFold.^36^ The residues affected by nonsynonymous mutations are highlighted in red (arrows). (G) Protein structure alignment of the C-terminal predicted sugar-binding domain of Amuc_1591 aligned with the crystal structure of the fucose kinase subunit of FKP.^39^ RMSD = 1.82 Å. Substitutions of residues located next to the active site bound by fucose-1-phosphate (white) are highlighted in red (arrows). (H) Agar plates containing 20  mM d-ara (left, control), supplemented with 5 mM L-fucose (middle), and 0.4% porcine mucin (right). (I) A model of the predicted function of Amuc_1591 as part of a biosynthetic pathway for the incorporation of l-fuc into LPS and the effect D-ara has on the activity of this predicted nucleotidylyltransferase.

To characterize the sensitivity of both *A. muciniphila* strains to D-ara, we cultured them in ADM supplemented with 0–20 mM of this sugar. Strikingly, we found that both strains are strongly inhibited by 100  µM d-ara, with JAX-AM001 subject to partial growth inhibition at 50  µM (Figure 2A, Figure S3A-B). The concentrations at which D-ara inhibits the growth of these two strains are one order of magnitude below that of typical carbon source supplementation into media. This high sensitivity suggests that D-ara is unlikely to impose a burden through metabolic or osmotic stress, suggesting instead that it interferes with a specific and sensitive physiological process.

As l-ara is a component of plant cell walls and far more abundant than D-ara in nature (Figure 2B),^40^ we hypothesized that it may also inhibit *A. muciniphila* growth. Surprisingly, we found that inhibition is specific to the D-form isomer, as l-ara does not alter the growth of either strain (Figure 2C). Furthermore, we found that the other five carbon, d-form sugars, such as d-ribose, do not affect *A. muciniphila* growth (Figure 1C−D, Figure S3C-D). Given that d-ara strongly inhibits growth in a stereo-specific manner, we hypothesized that it disrupts an essential biochemical process in *A. muciniphila*.

### Inactivation of a fucose-nucleotidyltransferase confers resistance to D-arabinose

To identify a genetic basis for the sensitivity of *A. muciniphila* to d-ara, we performed a screen for mutations that suppress this growth inhibition. First, we inoculated both strains into ADM supplemented with 0.1  mM d-ara. This d-ara concentration is slightly permissive to growth, which we reasoned would improve the likelihood of recovering spontaneous suppressor mutants compared to fully inhibitory concentrations (>0.5  mM) (Figure S3A-B). We then passaged the cultures twice into progressively higher concentrations of d-ara before plating on ADM supplemented with 20 mM D-ara to select for resistant colonies. From these plates, we isolated six BAA-835 and six JAX-AM001 spontaneous escape mutants and confirmed that they are capable of robust growth on 20  mM d-ara (Figure 2D). We sequenced the genomes of each mutant for comparison to the respective available reference genomes. Although we identified 48 mutations across the 12 escape mutants (Supplemental Data 1), all the mutants carried mutations in a single gene, *Amuc_1591* (Figure 2D). Notably, we identified three distinct *Amuc_1591* alleles across these strains (two non-synonymous substitutions and one insertion), consistent with independent adaptive events. In contrast, other mutations shared across all mutants were likely present in our parental strain and therefore considered background. Given the high genomic similarity between BAA-835 and JAX-AM001 (Figure S4), the repeated emergence of mutations in the same locus across both strains is unlikely to have arisen by chance.^41^ Instead, this pattern indicates that these alleles confer a common adaptive benefit under d-ara selection. We therefore focused on understanding the function of the protein encoded by *Amuc_1591* (Figure 2E).

Amuc_1591 is predicted to encode a nucleotidyltransferase. Two of our mutant alleles carry mutations encoding V176G and G326E substitutions in the predicted protein product, and the third encodes a frameshift resulting in a premature stop codon (Figure 2E). We used protein modeling to predict the Amuc_1591 structure, identify conserved domains, and compare the homology to available crystal structures (Figure 2F and S5A-D). Amuc_1591 consists of two domains connected by a flexible linker (Figure 2F). The *N*-terminal domain exhibits strong homology to adenylyl- and cytidylyltransferase domains known to transfer the corresponding nucleoside diphosphate (NDP) onto substrates bound by the C-terminus (Figure S5C). The C-terminal domain exhibits similarity to sugar kinases, including fucose and galactose kinases (Figure S5D). Alignment with a fucokinase (PDB: 9IIP^39^ revealed a structurally-related binding pocket (Figure 2G), with many fucose-1-phosphate coordinating residues conserved (Figure S5E). Based on these findings, we hypothesized that Amuc_1591 is a fucose-nucleotidylyltransferase.

The sugars L-fuc and d-ara are structurally similar (Figure 2B). Considering the rarity with which D-ara occurs in nature, we hypothesized that Amuc_1591 may non-selectively transfer NDP to D-ara, resulting in the accumulation of nucleoside diphosphate-d-ara (NDP-d-ara). Consistent with this hypothesis, the V176G and G326E substitutions are positioned near the Amuc_1591 binding pocket, where they are likely to have allosteric effects that may occlude d-ara (Figure 2F-G). The accumulation of aberrant NDP-d-ara intermediates could create toxicity by reducing nucleoside triphosphate (NTP) pools.^42^ Alternatively, NDP-d-ara may disrupt downstream biological processes normally involving l-fuc.

*A. muciniphila* encodes fucosidases capable of cleaving terminal l-fuc residues that cap MUC2 glycans.^25^ We therefore hypothesized that mucin supplementation would mitigate the inhibitory effects of d-ara due to the presence of l-fuc in the media. Indeed, we observed that supplementation with 0.4% (w/v) porcine mucin or 5  mM l-fuc into ADM restored normal growth (Figure 2H). These results support our conclusion that Amuc_1591 is a fucose-nucleotidyltransferase that interacts non-specifically with D-ara, resulting in growth inhibition.

Nucleotidylyltransferases are often involved in the synthesis of cell surface polysaccharides, glycoproteins, and other cell envelope/wall-associated glycans.^43^ Indeed, *A. muciniphila* incorporates l-fuc into the core oligosaccharides of lipopolysaccharide (LPS) molecules that comprise the outer leaflet of the outer membrane (OM).^44^ LPS glycans are essential, providing barrier function, rigidity, and stability to the OM.^45^ ^,^ ^46^ In both strains of *A. muciniphila*, Amuc_1591 is predicted to be encoded in the same transcriptional unit as *murJ* (Figure 2E), which encodes an essential lipid-II flippase required for cell wall biosynthesis.^47^ Together, this genetic, genomic, and metabolic evidence suggests that Amuc_1591 encodes a previously uncharacterized fucose-nucleotidyltransferase and may be involved in OM biosynthesis (Figure 2I). More broadly, these findings underscore that mucin-derived sugar scavenging in *A. muciniphila* supports essential biosynthetic processes, rather than serving exclusively as a source of carbon for metabolism.

### Glycosylation-derived monosaccharides inhibit early growth of *A. muciniphila*

**Figure 3.**
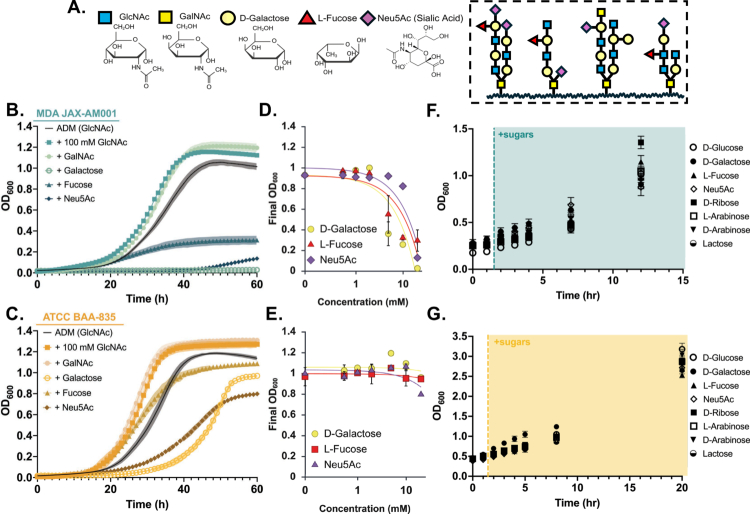
Elevated concentrations of free mucin-glycosylation sugars delay the lag phase and inhibit the growth of *A. muciniphila* in a strain-specific manner. (A) Structure of sugars that comprise mucin glycans and representative intestinal mucin glycan structures. (B, C) Growth of strains JAX-AM001 (B, teal) and BAA-835 (C, yellow) in media containing glycan-derived monosaccharides, measured in biological triplicate. (D, E) Dose‒response curves for the growth (final OD_600_) of strains JAX-AM001 (D) and BAA-835 (E) supplemented with inhibitory glycan sugars. (F, G) Exponential growth data of strains JAX-AM001 (F, teal) and BAA-835 (G, yellow). The addition of the respective sugar to cultures is indicated by the dotted line. Time points were recorded in biological triplicate.

Like D-ara, d-gal inhibits the growth of both BAA-835 and JAX-AM001, with strain-specific severity that is most pronounced in JAX-AM001 (Figure 1C, D). As d-gal is a core component of GI mucins,^1^ we next examined the effects of other glycan-derived monosaccharides on *A. muciniphila* growth (Figure 3A). Here, we cultured each strain in ADM supplemented with 20  mM GalNAc, l-fuc, or Neu5Ac and compared their growth to that of the strains supplemented with d-gal. Consistent with previous reports,^23^ both strains grow faster in media containing GlcNAc and GalNAc relative to GlcNAc alone (Figure 3B, C). Interestingly, Neu5Ac completely inhibits the growth of JAX-AM001, similar to d-gal, while l-fuc causes slow growth (T_d_ = 8 h) and a low final density (OD_600_ = 0.2) (Figure 3B). We observed a greater than 50% reduction in the final culture optical density in as low as 2  mM d-gal, 5  mM l-fuc, and 10  mM Neu5Ac, respectively (Figure 3D, Figure S6A–C). These results suggest that JAX-AM001 is most sensitive to d-gal and l-fuc, even when these sugars are present only as a minor component of ADM. Consistent with its tolerance of the dietary sugars we tested, the growth of BAA-835 was less impacted by these mucin-derived sugars (Figure 3C). The addition of d-gal or Neu5Ac (>10 mM) to BAA-835 cultures extends the lag phase (~20 h and ~26 h vs. ~14 h) and slows growth (T_d_ = 6.0 h and T_d_ = 7.4 h vs. T_d_ = 4.6 h) (Figure 3E, Figure S6D–F). In contrast, l-fuc (20  mM) did not have a major effect on BAA-835 growth, although cultures appeared to enter the stationary phase earlier (Figure 3C, Figure S6E). At concentrations below 5 mM, both l-fuc and d-gal modestly improved growth, with cultures entering the exponential phase earlier and reaching a higher final cell density (Figure S6A-E). Improved growth in less than 5 mM d-gal and l-fuc is consistent with previous evidence suggesting that BAA-835 has some capacity to utilize these sugars.^23-25^ To test whether these effects are specific to sugar identity rather than osmotic or other general physiological stress, we increased the GlcNAc concentration to 100  mM, which instead enhanced growth of both strains (Figure 3B, C).

These results highlight a conserved phenomenon where elevated concentrations of mucin-derived sugars predominantly prolong the lag phase. This effect might reflect a transient metabolic imbalance that arises when bacteria encounter high concentrations of mucin-derived sugars during the lag phase, such as the rapid accumulation of sugar‒phosphate intermediate, which reduces already limited ATP pools, or the disruption of redox balance before central metabolism is fully engaged.^42^ To determine if this sensitivity is limited to the lag phase, we allowed each strain to reach the mid-log phase before supplementing each growth-inhibiting sugar that we identified. None of these supplemented sugars had a negative effect on the growth of log-phase cultures of JAX-AM001 (Figure 3F) or BAA-835 (Figure 3G). We conclude that mucin-derived sugars inhibit the ability of *A. muciniphila* to transition from the lag into exponential growth phase and next sought to identify the genetic basis of this sensitivity.

### Galactokinase and succinyl-CoA synthetase mutations improve *A. muciniphila* growth on D-galactose

**Figure 4.**
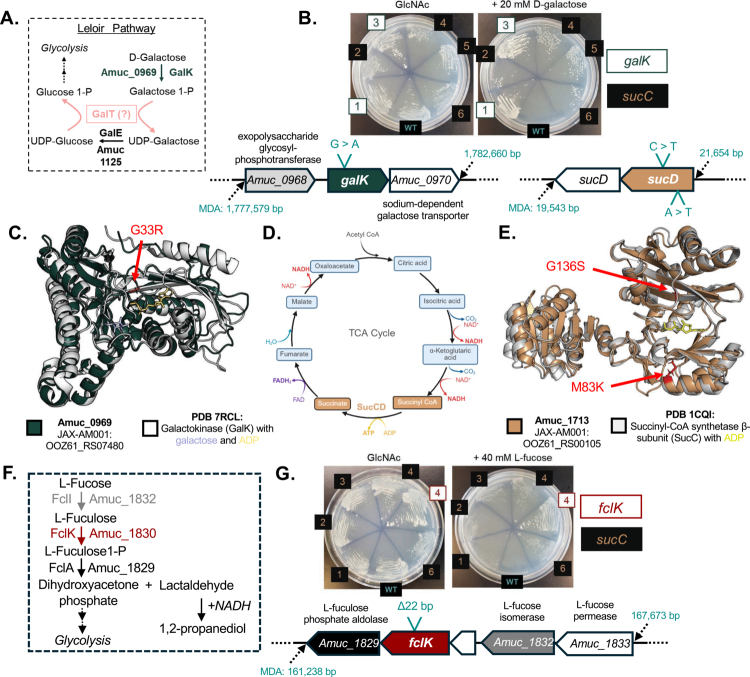
d-gal and l-fuc suppressors highlight central carbon and carbon overflow metabolism. (A) Pathways for d-gal utilization (Leloir pathway, KEGG: amu00052). (B) Growth of isolated mutants (1–6) relative to the parental strain (WT) with or without d-gal. The genomic regions display the location of point mutations identified in d-gal suppressor mutants. (C) The predicted protein structure model of GalK (green, Amuc_0969) aligned to the crystal structure of GalK (white, PDB: 7CRL.^49^ RMSD = 0.82 Å. The residue affected by a common nonsynonymous mutation is highlighted in red (arrow). (D) Diagram of the tricarboxylic acid (TCA) or citric acid cycle (KEGG: amu00020). (E) The predicted protein structure model of SucC (Amuc_1713) aligned with the crystal structure of SucC (PDB: 1CQI.^50^ RMSD = 1.41 Å. The residues affected by common nonsynonymous mutations are highlighted in red (arrows). (F) The pathway for l-fuc utilization (KEGG: amu00051). (G) Growth of isolated mutants (1–6) relative to the parental strain (WT) with or without l-fuc. The location of a 22 bp deletion identified in a suppressor mutant was mapped to *fclK* within the predicted fucose-utilization operon encoded by *A. muciniphila.*

In bacteria, d-gal is typically catabolized through the Leloir pathway (Figure 4A). However, *A. muciniphila* appears to lack a complete Leloir pathway, as it is missing a predicted homolog of *galT*, and no suitable candidate was detected by BLAST or domain-based searches (Figure S7A). This result led us to hypothesize that high concentrations of environmental d-gal cause the accumulation of galactose-1-phosphate via GalK function. The accumulation of galactose-1-phosphate has been shown to be toxic in other bacteria due to depletion of ATP pools,^42^ and this toxicity can be suppressed by GalK inactivation.^48^ We therefore sought to identify mutations that suppress d-gal-mediated growth inhibition.

We focused our screen on JAX-AM001, which displayed the most pronounced growth inhibition in the presence of d-gal (Figure 3). By first culturing JAX-AM001 in liquid media containing 10  mM d-gal, we isolated six spontaneous escape mutants capable of growth on plates containing 20  mM d-gal (Figure 4B). Whole-genome sequencing revealed that two of the six mutants contained a non-synonymous point mutation within *Amuc_0969* encoding a G33R substitution in the predicted protein product (Figure 4B, Supplemental Data 2). Consistent with our expectations, the *Amuc_0969* locus is predicted to encode the galactokinase GalK. To assess the functional impact of this non-synonymous mutation, we generated a structural model of Amuc_0969 (Figure S7B-C) and aligned it with the crystal structure of a GalK homolog from *Lactococcus lactis* (PDB: 7RCL.^49^ This analysis revealed that G33R lies near the putative ATP-binding pocket (Figure 4C, Figure S7D). The substitution of glycine for arginine at this site is predicted to result in steric clashes that negatively impact kinase function (Figure S7D). Combined, our data suggest that G33R reduces GalK activity, thereby limiting the toxic accumulation of galactose-1-phosphate in *A. muciniphila* grown in D-gal.

The remaining four suppressor mutants encode point mutations within *Amuc_1713* (Figure 4B), which is predicted to encode the *β* subunit of succinyl-CoA synthetase (SucC). The SucCD (Amuc_1713/Amuc_1712) complex is responsible for the fifth step of the TCA cycle, converting succinyl-CoA into succinate, regenerating CoA, and producing ATP (Figure 4D). The two point mutations we identified encode substitutions at M83K and G136S. To determine how these substitutions may affect SucC function, we again used protein modeling to predict the structure of Amuc_1713 and compare it with available crystal structures (Figure 4E, Figure S8). These substitutions are located proximal to residues that directly coordinate ADP and Mg^2+^ (PDB: 1CQI),^50^ and are predicted to affect charge, steric bulk, and flexibility near the active site (Figure S8D). Thus, we predict that the M83K and G136S substitutions likely interfere with SucC ADP-binding and reduce SucCD enzymatic activity.

As *A. muciniphila* is predicted to lack a complete Leloir pathway, the phosphorylation of d-gal by GalK likely creates a futile ATP-consuming reaction that leads to the toxic accumulation of gal-1-phosphate and perturbs the energy balance during the lag phase. While reducing SucC activity would also be expected to lower ATP production via the TCA cycle, our results suggest that growth inhibition arises from metabolic imbalance rather than ATP limitation alone. We propose that unproductive GalK activity drives compensatory TCA flux, amplifying redox and energy stress during early growth. Mutations that dampen SucC activity likely reduce this excess flux, slowing metabolic throughput and stabilizing energy and redox balance, thereby permitting growth initiation under otherwise inhibitory conditions.

### Inactivation of fuculokinase rescues JAX-AM001 growth on L-fucose

In *A. muciniphila*, l-fuc enters glycolysis via a four-step pathway producing glyceraldehyde-3-phosphate while secreting 1,2-propanediol and requiring energy investment of ATP and NADH (Figure 4F). To identify the genetic basis of l-fuc sensitivity, we screened for spontaneous suppressor mutants capable of overcoming l-fuc growth inhibition. Because l-fuc does not completely inhibit growth at 20  mM (Figure 3B), we performed our screen at 40 mM l-fuc. We isolated multiple escape mutants capable of growth in the presence of 40  mM l-fuc (Figure 4G). Of the six mutants analyzed, whole-genome sequencing revealed one suppressor strain carrying a 22 bp deletion in *Amuc_1830,* which is predicted to encode the fuculokinase FclK (Figure 4G, Supplemental Data 3). The inactivation of FclK, which catalyzes the first committed step in L-fuc catabolism, suggests that inhibition arises from energy expenditure and the accumulation of fucose-1-phosphate early in the pathway. By preventing this initial kinase reaction, suppressor mutants likely conserve ATP and avoid sugar‒phosphate stress during the lag phase, restoring balanced metabolism and enabling normal growth in the presence of excess l-fuc.

Interestingly, we only observed FclK inactivation in one of the six mutants we sequenced. The remaining five had *sucC* mutations similar to those identified in our d-galactose suppressor strains (Figure 4D, Supplemental Data 3). In the gut, anaerobic bacteria rely predominantly on glycolysis or fermentation rather than a fully oxidative TCA cycle owing to the lack of appropriate terminal electron acceptors.^51^ Consequently, high concentrations of l-fuc are likely shunted through the TCA cycle owing to carbon overflow. The recurrence of *sucC* mutations in l-fuc tolerant strains suggests that stress can be overcome by dampening central carbon metabolism and reducing flux through the TCA cycle. By limiting excess flux through an inefficient TCA cycle, these mutations likely protect *A. muciniphila* from redox imbalance and energy stress during the lag phase, stabilizing growth under high-sugar conditions. Collectively, suppressors of both d-gal and l-fuc sensitivity reveal that growth inhibition by mucin-derived sugars arises from energetic constraints and metabolic bottlenecks feeding into glycolysis and the TCA cycle.

## Discussion

*A. muciniphila* occupies a critical niche in the gut as a mucin-degrading specialist, emerging as a key symbiont with an increasingly recognized role in GI health.^52^ This specialization tightly links *A. muciniphila* physiology to the integrity and composition of the mucosal barrier, which is evident in its evolved auxotrophy for mucin-derived sugars and amino acids^23^ and its reliance on MUC2 epitopes for adhesion and colonization of the colon mucosa.^53^ Despite its broad capacity to release monosaccharides that comprise mucin, this bacterium was reported to selectively metabolize amino sugars (GlcNAc and GalNAc), with only limited utilization of d-gal or l-fuc and no use of Neu5Ac.^23-25^ However, the utilization of d-gal and l-fuc in any capacity appears to be strain specific, as even low concentrations impair JAX-AM001 growth (Figure S6), highlighting strain-level variation in sugar tolerance and metabolism. Nonetheless, to access core glycan sugars and peptide stems, these three sugars must be enzymatically processed during mucin degradation. Owing to their limited benefits for growth, they are instead released into the environment where they are proposed to support the growth of co-occurring taxa.^5^ ^,^ ^6^ ^,^ ^25^ Here, we expand on the role of these sugars, finding that elevated concentrations of d-gal, l-fuc, and to a lesser extent, Neu5Ac, specifically inhibit or delay the growth of *A. muciniphila*.

Inhibition by these mucin-derived sugars is specific to the lag phase, suggesting that they affect metabolic processes essential for the transition from dormancy to active growth. During lag, both ATP reserves and enzyme pools are low, reducing the overall metabolic buffering capacity and leaving bacteria more susceptible to metabolic bottlenecks and stress.^54^ In combination with the high energetic cost of initiating mucin-degradation programs and rebuilding biosynthetic capacity, even transient imbalances in carbon metabolism may disrupt redox and energy homeostasis in *A. muciniphila*, delaying entry into exponential growth. This vulnerability is best modeled by sensitivity to mucin-derived sugars that enter incomplete (d-gal) or energetically costly pathways (l-fuc). These sugars impose the strongest constraints through predicted futile ATP expenditure and redox imbalance associated with partial catabolic routes or carbon overflow, respectively. In contrast, Neu5Ac, which cannot be metabolized, produces the mildest inhibition, pointing to a non-metabolic source of stress. Together, these observations reinforce a model in which GlcNAc and/or GalNAc are strongly favored carbon sources. More broadly, they suggest that sensitivity to mucin-derived sugars reflects a metabolic checkpoint, where growth initiation depends on balancing energy and redox demands rather than on carbon availability alone.

**Figure 5.**
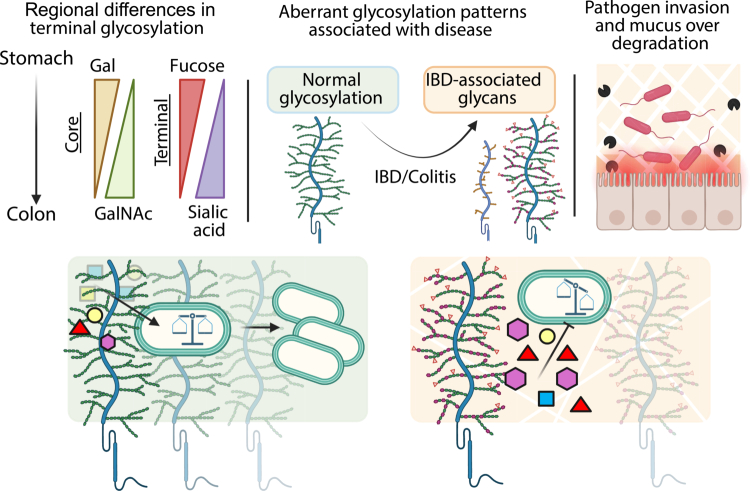
The composition and integrity of mucin likely influence local concentrations of environmental, host-derived saccharides and affect the central carbon metabolism of *A. muciniphila*. (Top) A model of the regional, disease, or pathogen-associated differences that influence mucin glycan composition and integrity throughout the gastrointestinal tract. (Bottom) Under normal conditions, *A. muciniphila* degradation of colonic mucin glycans (MUC2) releases terminal sugars that are consumed primarily by other mucus-associated microbes. Core amino sugars (GlcNAc and GalNAc) are preferentially consumed by *A. muciniphila*. On the other hand, conditions that affect mucin composition or integrity are predicted to result in increased concentrations of inhibitory sugars (such as d-gal and l-fuc). This imbalance in localized sugar concentrations is predicted to result in sugar toxicity and metabolic stress, thereby inhibiting colonization dynamics within the gut.

Overall, these findings raise a key evolutionary question: why has *A. muciniphila* retained sugar kinases that initiate pathways it cannot fully execute? The persistence of GalK despite the loss of GalT and the high energetic cost of l-fuc catabolism suggests that these pathways may serve regulatory rather than purely catabolic roles. Given the metabolic strain imposed by d-gal and l-fuc during the early phases of growth, the activity of these kinases may also function as metabolic “brakes” by indirectly reporting on mucin integrity or composition, ensuring that outgrowth only proceeds when preferred core sugars become available. This conclusion aligns with broader work demonstrating that bacterial fitness under stress is often governed not by nutrient availability alone, but how carbon flux, redox balance, and biosynthetic demand are coordinated during specific physiological states.^54-56^ Thus, pathways that appear inefficient from a catabolic perspective may instead serve to reinforce niche specialization by coordinating growth with mucin composition or cohabitating microbes. More broadly, retention of these pathways may generate intermediates that serve unknown roles, analogous to other seemingly futile cycles with established signaling, buffering, or regulatory functions.^55^ Therefore, we hypothesize that mucin-derived sugars and glycan composition likely affect the colonization patterns of *A. muciniphila* in the GI tract (Figure 5). Although mucin glycans are encountered as complex structures *in vivo*, their stepwise degradation and limited diffusion within the mucus layer likely generate localized and transient enrichment of individual monosaccharides, particularly during active mucin turnover or barrier disruption. Consistent with this, the inhibitory effects we observe are restricted to the lag phase, suggesting that even transient exposure to specific sugars can influence growth initiation before metabolic equilibrium is established.

Consistent with this idea, mucin glycan landscapes vary substantially along the GI tract, between hosts, and in response to inflammation or infection.^3^ ^,^ ^30^ For example, the colonic mucus layer in mice is substantially thinner than that in humans,^49^ creating differences in mucin availability, glycan turnover, and the local concentrations of released sugars experienced by mucolytic bacteria. Indeed, we found that the mouse-derived strain is more sensitive to mucin-derived sugars than the human isolate (Figure 3). These results hint at host-specific adaptation of these metabolic pathways as sensors to properly assess local mucosal conditions. Likewise, differences in mucin glycan composition along the GI tract, where GalNAc and Neu5Ac become more abundant towards the colon while both d-gal and l-fuc decline,^10^ ^,^ ^12^ mirror the preferential localization of *A. muciniphila* to the distal gut.^57^ This raises the possibility that gradients in glycan composition act as environmental cues. Such gradients, in conjunction with these encoded metabolic sensors, may help *A. muciniphila* position itself in regions where metabolic demands and sugar composition are compatible with its physiology as it traverses the GI tract. Similarly, aberrant mucin glycosylation patterns that mark chronic inflammation, including abundant d-gal cores and heavily sialylated (Neu5Ac) glycans,^3^ ^,^ ^58^ ^,^ ^59^ correlate with a reduced abundance of *A. muciniphila.* ^21^ ^,^ ^22^ Pathogen-driven changes in mucus integrity, such as antibiotic-associated *Clostridioides difficile* infections,^60^ expression of mucin-degrading glycosidases by opportunistic pathogens^6^ or epithelial invasion by *Salmonella* Typhimurium,^61^ likely increase exposure to free glycan sugars while compromising barrier function.^60^ These shifts could elevate inhibitory monosaccharide concentrations at the mucosal interface due to over degradation of MUC2 glycans and restrict *A. muciniphila* colonization, offering a possible explanation for the observed decline in abundance during infection.^62^

Beyond host glycan composition, these metabolic constraints may also structure interactions between *A. muciniphila* and co-occurring taxa, reinforcing its role in metabolic exchange within the gut microbiota. *A. muciniphila* appears to prioritize metabolic investment in mucin cores, extracting GlcNAc and GalNAc, while releasing energetically expensive or non-metabolizable sugars to support cross-feeding partners. This conclusion aligns with previous co-culturing experiments where *A. muciniphila* supports the growth of important commensals such as *Anaerostipes caccae, Eubacterium hallii*, and *Faecalibacterium prausnitzii*, which are unable to process intact mucin.^25^ ^,^ ^63-65^ These interspecies metabolic interactions, combined with the inherent differences in the gut microbiota composition between hosts,^66^ may also help explain the strain-specific difference we observed. For example, a higher abundance of naturally occurring cross-fed taxa may buffer *A. muciniphila* from the inhibitory effects of these sugars. If these taxa are more prevalent in mice, this may explain why JAX-AM001 has not evolved to be more tolerant to higher concentrations. More speculatively, it is also possible that the abundance of cross-fed taxa differs between humans as well, based on their individual gut community structures. This, in turn, may contribute to which strain(s) or even phylogroup(s) of *A. muciniphila* colonize an individual at any given time, as evident by the growing number of unique strains isolated from healthy human donors.^67^ ^,^ ^68^

While the sensitivity to mucin-derived monosaccharides appears to be conserved between BAA-835 and JAX-AM001, these two strains exhibit different responses to dietary sugars. Generally, the human-derived strain (BAA-835) tolerates or benefits from common diet-derived carbohydrates, such as d-glucose, d-fructose, and lactose, while the mouse-derived strain (JAX-AM001) is inhibited by them. This trend holds true for mucin-derived sugars, where the human strain appears to benefit from low levels of d-gal and l-fuc, consistent with previous reports,^23-25^ whereas JAX-AM001 was highly sensitive. These physiological differences further support a model that reflects adaptation to host-specific nutrient environments (human vs. murine diets) and glycan architectures (colonic mucus density). Consistent with these observations, the human-isolate BAA-835 appears to have evolved a broader tolerance for fluctuating dietary nutrient inputs, enabling more efficient metabolism of diverse sugars. This may also explain the differences in l-fuc tolerance, where BAA-835 is better adapted to tolerate carbon flux and energy investment requirements via the complete l-fuc catabolic pathway.

In summary, our findings reveal that mucin-derived sugars function not only as nutrients but also as metabolic stressors whose impact depends on the strain background, metabolic context, and growth phase. These findings establish a framework linking mucin-derived sugars to growth regulation, while highlighting key areas for future study. Efforts extending this work to more complex and physiologically relevant systems, including complex glycans, metabolite profiling, and *in vivo* or community-based models will be essential to support these findings. Critically, future efforts to resolve carbon flux through these pathways, validate the effects of individual mutant alleles, further elucidate cross-feeding relationships, and determine how transient sugar exposure shapes growth in the native mucosal environment are needed to best contextualize these physiological responses. Additionally, expanding this analysis across additional *A. muciniphila* strains will further clarify how conserved these metabolic strategies are and how they contribute to host-specific adaptation and colonization. Nonetheless, by identifying novel carbon-overflow bottlenecks and suppressor mutations that alleviate sugar-induced stress, we show that *A. muciniphila* has evolved metabolic brakes that possibly prevent the onset of growth in environments with aberrant or degraded mucin-glycans. These strain-specific responses underscore the influence of host diet, anatomy, and glycan composition in shaping microbial physiology and niche specialization. Our analyses can be repeated for newly isolated *A. muciniphila* strains identified in future studies, helping to resolve genetic and phenotypic diversity amongst strains, informing strain-selective approaches for modulating mucosal health and the deployment of engineered probiotics.^69^

## Supplementary Material

Supplementary Material Supplemental_data.xlsx

Supplementary Material SI_Appendix.pdf

## Data Availability

The authors confirm that the data supporting the findings of this study are available within the article and/or its supplementary materials.
