# Supplementary material for: Mucin-derived sugars act as metabolic brakes controlling growth initiation in Akkermansia muciniphila
Source: Gut Microbes. 2026 Jun 26;18(1):2691334. doi: 10.1080/19490976.2026.2691334 (PMC13313206; doi:10.1080/19490976.2026.2691334)
Supplement: Supplementary Material — SI_Appendix.pdf [file KGMI_A_2691334_SM5500.pdf]

Supporting Information for

Mucin-derived sugars act as metabolic brakes controlling growth initiation in *Akkermansia muciniphila*

Bryan D. Lakey*, Katherine J. Wozniak, Robert A. Britton, and Jeffrey J. Tabor*

*Email: blakey@mdanderson.org; jeffery.tabor@rice.edu

This PDF file includes:

Figures S1 to S8

Tables S1

Legends for Datasets S1 to S5

SI References

Other supporting materials for this manuscript include the following:

Datasets S1 to S5

Figures

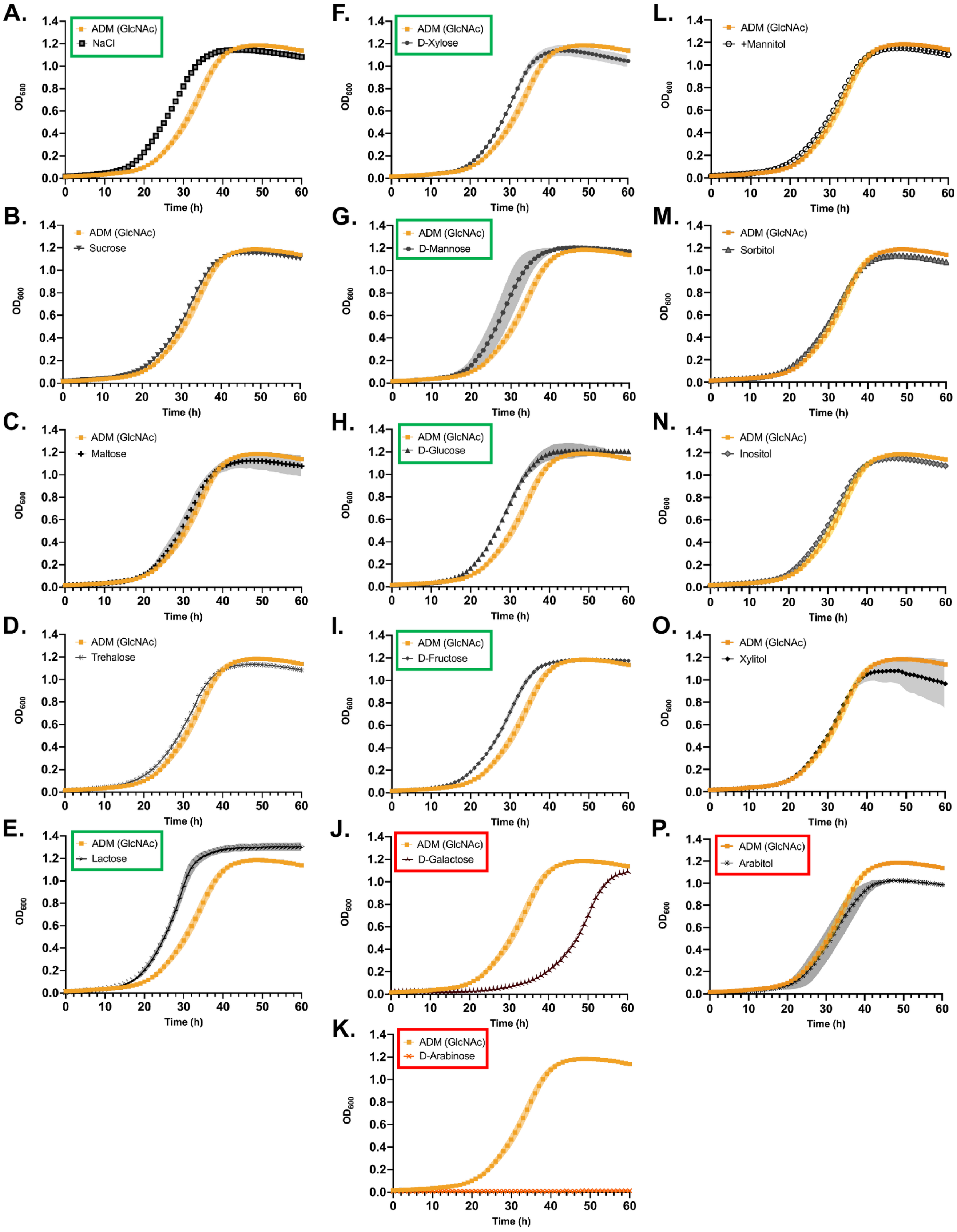
A.
ADM (GlcNAc)
NaCl
B.
ADM (GlcNAc)
Sucrose
C.
ADM (GlcNAc)
Maltose
D.
ADM (GlcNAc)
Trehalose
E.
ADM (GlcNAc)
Lactose
F.
ADM (GlcNAc)
D-Xylose
G.
ADM (GlcNAc)
D-Mannose
H.
ADM (GlcNAc)
D-Glucose
I.
ADM (GlcNAc)
D-Fructose
J.
ADM (GlcNAc)
D-Galactose
K.
ADM (GlcNAc)
D-Arabinose
L.
ADM (GlcNAc)
+Mannitol
M.
ADM (GlcNAc)
Sorbitol
N.
ADM (GlcNAc)
Inositol
O.
ADM (GlcNAc)
Xylitol
P.
ADM (GlcNAc)
Arabitol
OD600
Time (h)

Supplemental Figure S1. ATCC BAA-835 Sugar and Sugar Alcohol Growth Curves. Growth data for strains ATCC BAA-835 grown in ADM (yellow) supplemented with 20 mM of various sugars (A-K) and sugar alcohols (L-P). Each growth condition was measured in triplicate. Green and red boxes indicate positive and negative effects on growth, respectively.

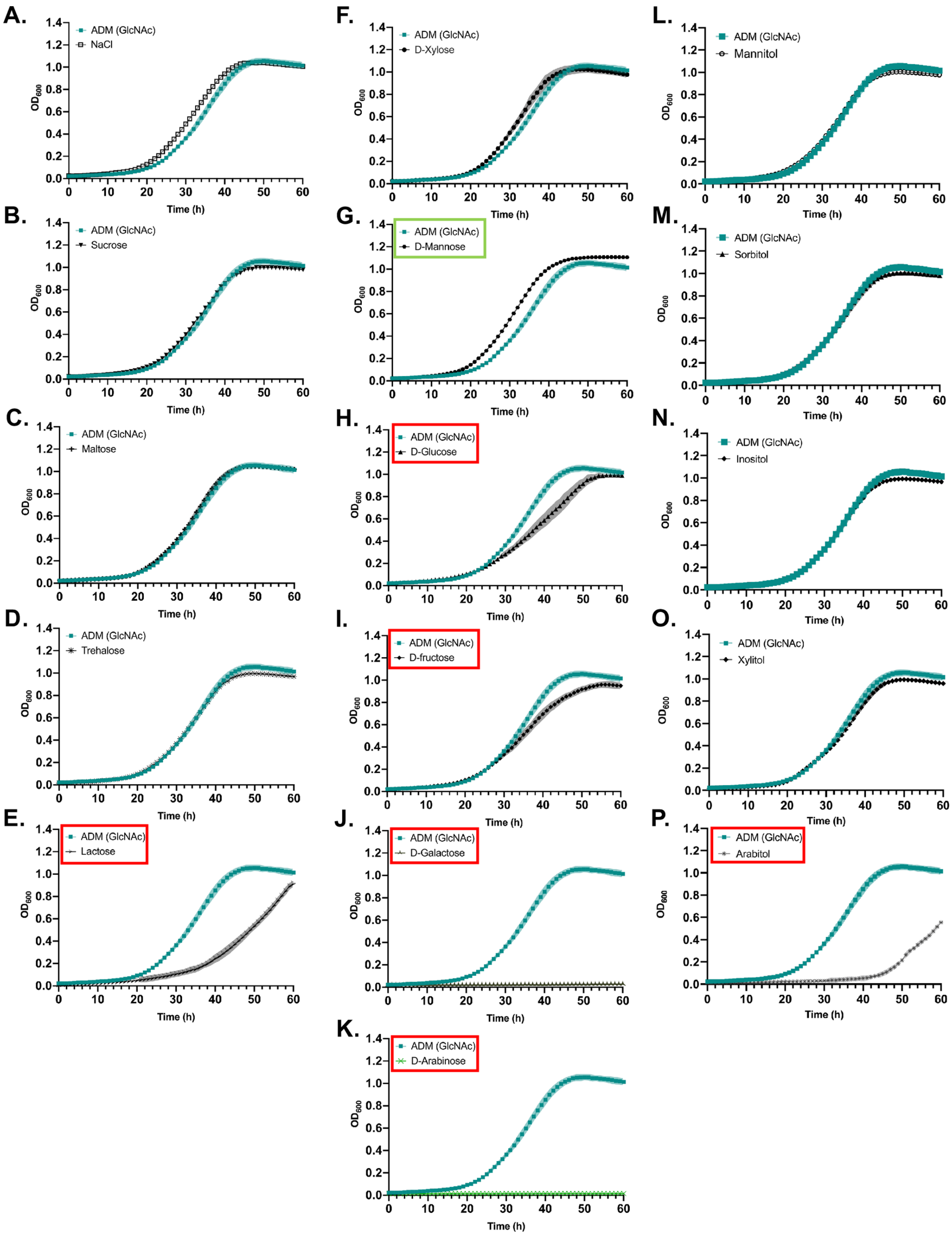
A.
ADM (GlcNAc)
NaCl
B.
ADM (GlcNAc)
Sucrose
C.
ADM (GlcNAc)
Maltose
D.
ADM (GlcNAc)
Trehalose
E.
ADM (GlcNAc)
Lactose
F.
ADM (GlcNAc)
D-Xylose
G.
ADM (GlcNAc)
D-Mannose
H.
ADM (GlcNAc)
D-Glucose
I.
ADM (GlcNAc)
D-fructose
J.
ADM (GlcNAc)
D-Galactose
K.
ADM (GlcNAc)
D-Arabinose
L.
ADM (GlcNAc)
Mannitol
M.
ADM (GlcNAc)
Sorbitol
N.
ADM (GlcNAc)
Inositol
O.
ADM (GlcNAc)
Xylitol
P.
ADM (GlcNAc)
Arabitol
OD600
Time (h)

Supplemental Figure S2. MDA JAX-AM001 Sugar and Sugar Alcohol Growth Curves. Growth data for strains MDA JAX-AM001 grown in ADM (teal) supplemented with 20 mM of various sugars (A-K) and sugar alcohols (L-P). Each growth condition was measured in triplicate. Green and red boxes indicate positive and negative effects on growth, respectively.

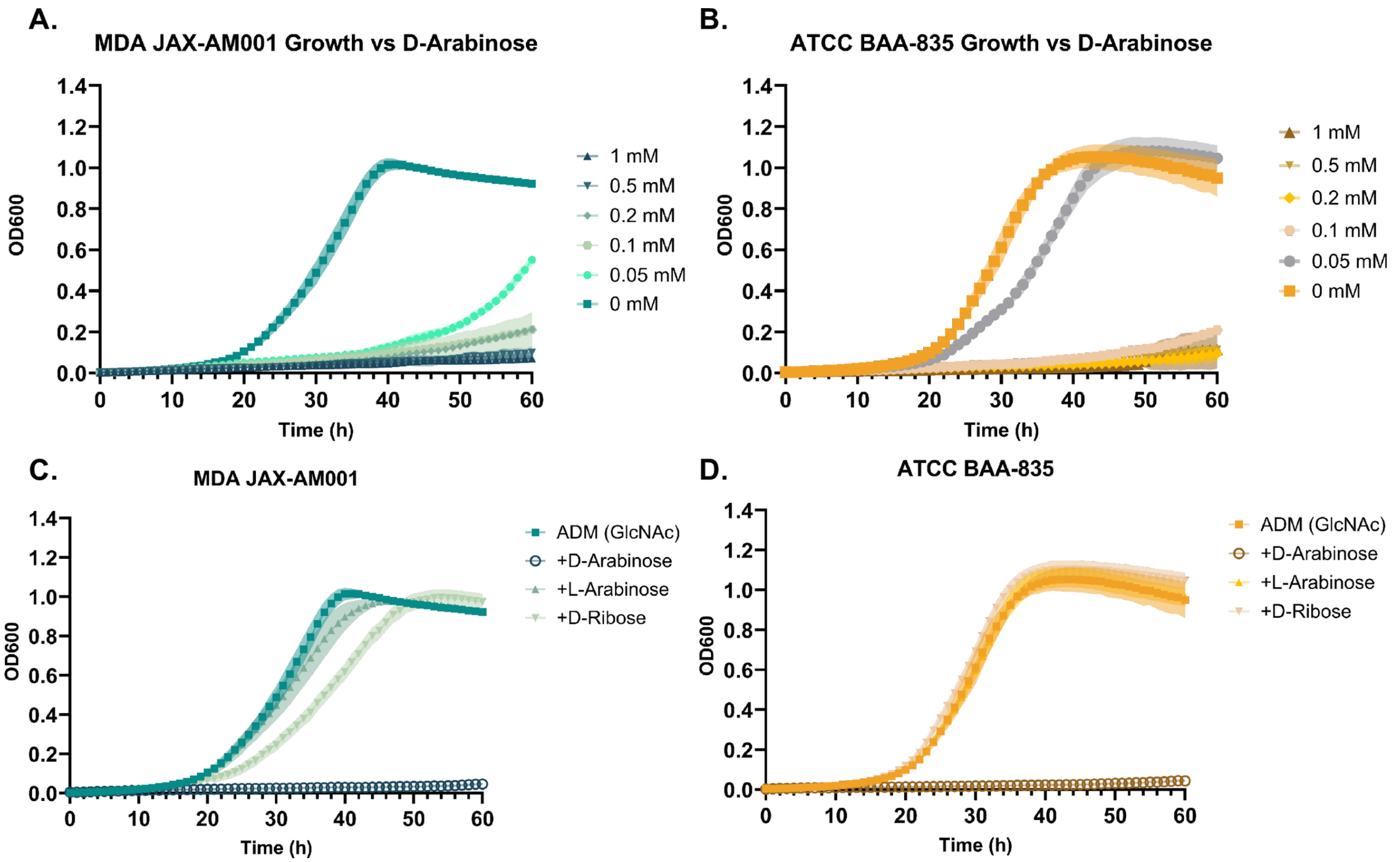

Supplemental Figure S3. Both strains of *A. muciniphila* are sensitized to low levels of D-arabinose in a minimal media. (A-B) Growth curves of strains JAX-AM001 (A) and BAA-835 (B) in media containing decreasing concentrations of D-arabinose. **(C-D)** Growth curves of strains JAX-AM001 (C) and BAA-835 (D) in media supplemented with five carbon sugars (20 mM).

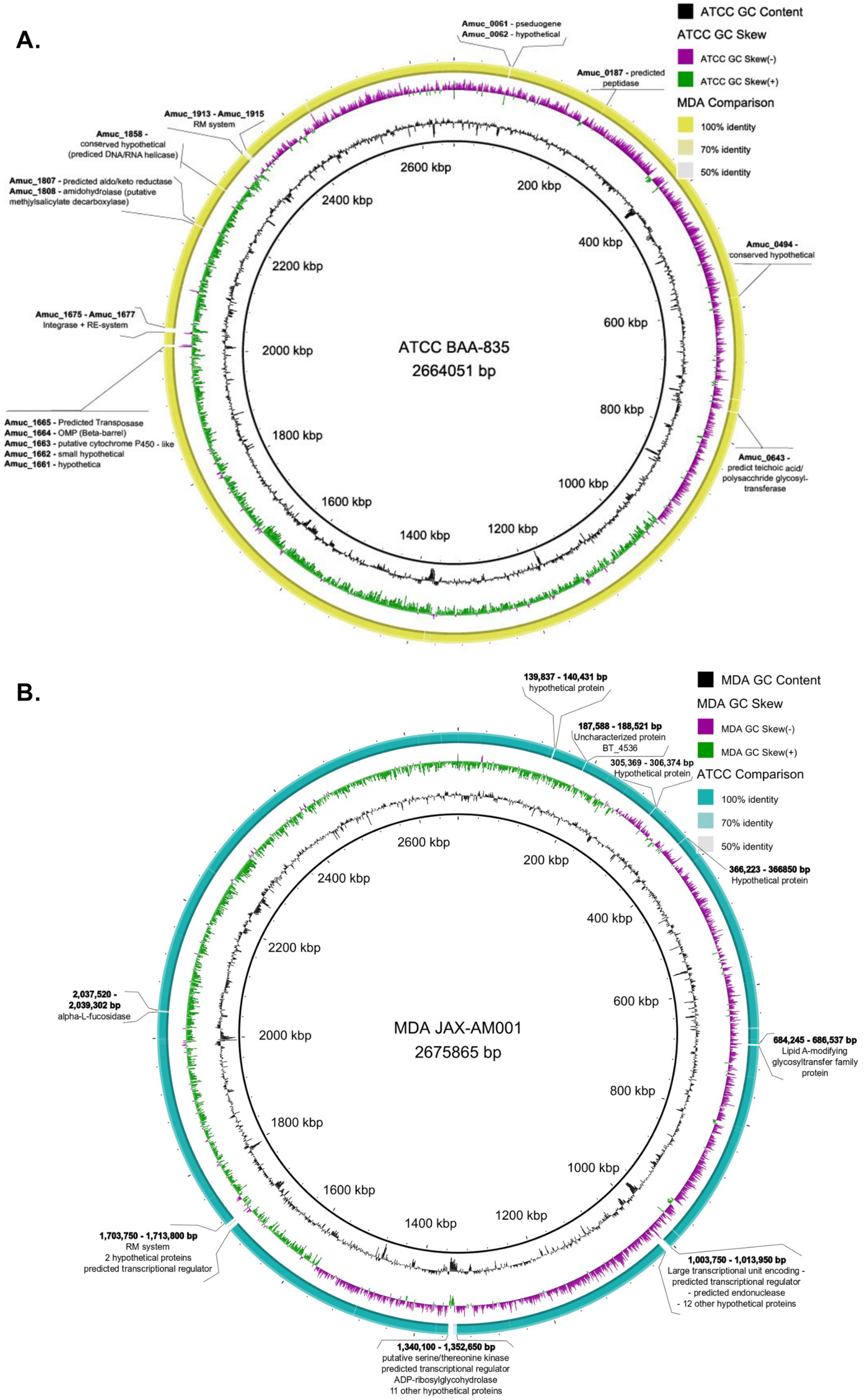
A.
ATCC GC Content
ATCC GC Skew
ATCC GC Skew(-)
ATCC GC Skew(+)
MDA Comparison
100% identity
70% identity
50% identity
Amuc_0061 - pseduogene
Amuc_0062 - hypothetical
Amuc_0187 - predicted peptidase
Amuc_1913 - Amuc_1915 RM system
Amuc_1858 - conserved hypothetical (prediced DNA/RNA helicase)
Amuc_1807 - predicted aldo/keto reductase
Amuc_1808 - amidohydrolase (putative methjylsalicylate decarboxylase)
Amuc_0494 - conserved hypothetical
Amuc_1675 - Amuc_1677 Integrase + RE-system
Amuc_1665 - Predicted Transposase
Amuc_1664 - OMP (Beta-barrel)
Amuc_1663 - putative cytochrome P450 - like
Amuc_1662 - small hypothetical
Amuc_1661 - hypothetica
Amuc_0643 - predict teichoic acid/ polysacchride glycosyl-transferase
ATCC BAA-835
2664051 bp
2600 kbp
200 kbp
2400 kbp
400 kbp
2200 kbp
600 kbp
2000 kbp
800 kbp
1800 kbp
1000 kbp
1600 kbp
1400 kbp
1200 kbp
B.
MDA GC Content
MDA GC Skew
MDA GC Skew(-)
MDA GC Skew(+)
ATCC Comparison
100% identity
70% identity
50% identity
139,837 - 140,431 bp hypothetical protein
187,588 - 188,521 bp Uncharacterized protein BT_4536
305,369 - 306,374 bp Hypothetical protein
366,223 - 366850 bp Hypothetical protein
2,037,520 - 2,039,302 bp alpha-L-fucosidase
684,245 - 686,537 bp Lipid A-modifying glycosyltransfer family protein
1,703,750 - 1,713,800 bp RM system 2 hypothetical proteins predicted transcriptional regulator
1,003,750 - 1,013,950 bp Large transcriptional unit encoding - predicted transcriptional regulator - predicted endonuclease - 12 other hypothetical proteins
1,340,100 - 1,352,650 bp putative serine/thereonine kinase predicted transcriptional regulator ADP-ribosylglycohydrolase 11 other hypothetical proteins
MDA JAX-AM001
2675865 bp
2600 kbp
200 kbp
2400 kbp
400 kbp
2200 kbp
600 kbp
2000 kbp
800 kbp
1800 kbp
1000 kbp
1600 kbp
1400 kbp
1200 kbp

Supplemental Figure S4. Comparison of MDA and ATCC genomes. Circular representations of the ATCC genome (A, yellow) and the MDA genome (B, teal) compared to one another. Genomic sequences are generally well conserved across these two genomes (outer ring). The white spaces represent high-level divergence between genomes and the genes encoded at each site are listed. Many of these sites correspond to hypothetical proteins, restriction modification systems, or represent genome rearrangements.

A.

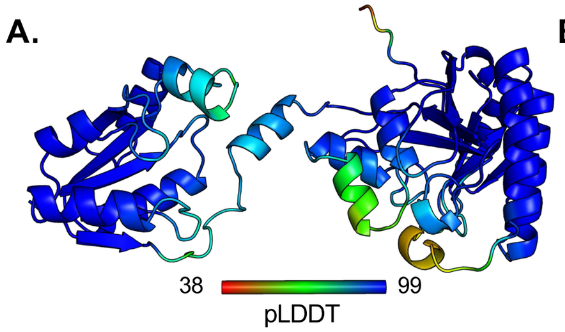

B.

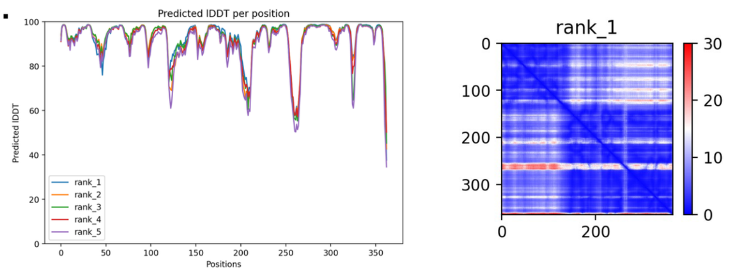

C. Amuc_1591
N-terminus (M1-T137):

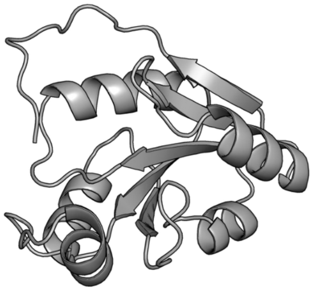

PDB ID	Z-score	RMSD	Aligned residues	Total residues	% ID	Description
5x9q	16.1	2.0	120	158	27	Putative Cytidylyltransferase
4xsv	14.3	2.1	115	306	26	Ethanolamine-Phosphate Cytidylyltransferase
9iyg	13	2.4	117	176	19	Phosphopantetheine Adenylyltransferase
3do8	11.9	2.8	115	1358	14	Phosphopantetheine Adenylyltransferase
4mvd	11.9	2.3	11	253	22	Choline-Phosphate Cytidylyltransferase
2r5w	10.2	2.4	110	345	13	Nicotinamide-Nucleotide Adenylyltransferase

D. Amuc_1591
C-terminus (L146-K363):

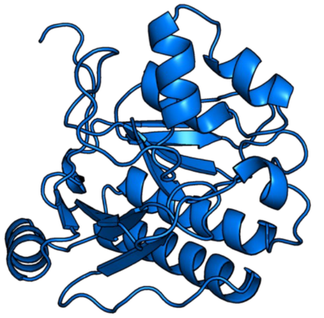

PDB ID	Z-score	RMSD	Aligned residues	Total residues	% ID	Description
9iit	18.7	2.6	208	950	22	L-Fucokinase/L-Fucose-1-P Guanylyltransferase
8tfo	18.2	2.5	202	309	20	Mevalonate Kinase
3k17	17.9	2.8	207	355	14	LIN0012 Protein
6teq	17.3	2.7	203	418	14	Galactokinase
5yys	15.9	2.8	209	811	19	L-Fucokinase, L-Fucose-1-P Guanylyltransferase
3v2u	15.4	2.7	201	516	16	Galactose/Lactose Metabolism Regulatory Protein G
4p52	15.0	2.8	194	315	15	Homoserine Kinase
4dxl	14.9	2.7	186	304	15	4-Diphosphocytidyl-2-C-Methyl-D-Erythitol Kinase

E.

```
(":" denotes aligned residue pairs of d < 5.0 A, "." denotes other aligned residues)
--------------------LRVDFAGGWLDVPRYA-RKGSYVVNCAITP---MV-SL--------------C-----------------------------------------------------------------EWPYEKRSGLGGSGAWAMLEGR---------
                    ::::::::::::::::: .:..:::::::::   :: :.              .                                                                 .::::::::::::::::::::::.
RKSTPKLDVYSDQIVWGRSPVRIDMAGGWTDTPPYSLYSGGNVVNLAIELNGQPPLQVYVKPCKDFHIVLRSIDMGAMEIVSTFDELQDYKKIGSPFSIPKAALSLAGFAPAFSAVSYASLEEQLKDFGAGIEVTLLAAIPAGSGLGTSSILASTVLGAINDFCGLA

--------DPVASELA--LGVGWQDPAVIAETGLCVWRSG----SSPVLDVKGT--GDF--LEGRMAILYTGEEHDTPKMADE-QR-DYVRISQSSLIARTGVLERNINTLAAGVALYYSVQLD-EG-MR----P-LPD-I-PNALAKKYLGGGYGGYALYLFPCRDD
        ::::::::  ::::::::::::::::::::::    ::::::::::  :::  .:::::::::::::         .. ::::::::::::::::::::::::::::::::::::: :: ::    : .:: : :::::::::::::::::::::::::::
WDKNEICQRTLVLEQLLTTGGGWQDQYGGVLQGVKLLQTEAGFAQSPLVRWLPDHLFTHPEYKDCHLLYYTGITR--------LHLNLLSEMKAHALDMNEAIQRGSFVEFGRLVGKTWEQNKALDSGTNPPAVEAIIDLIKDYTLGYKLPGAGGGGYLYMVAKDPQA

RDQAVKDNP-------A-MKRVEPYCRQ-LFK----
:::::::::       : :::::::::. .
AVRIRKILTENAPNPRARFVEMTLSDKGFQ--VSRS
```

Identical amino acids
Similar amino acids
Different amino acids

Supplemental Figure S5. Structure modeling of Amuc_1591. (A) Relaxed model of Amuc_1591 colored by pLDDT values (per residue measurement of local confidence, local distance difference test). (B) Plot of pLDDT score over the length of each of the 5 models that ColabFold generated (1). The highest ranked structural predict was used in this analysis. Position alignment error (PAE) plot for the highest ranked structure is shown (right). PAE is a measure of confident in the relative position of two residues within the predicted structure (colored by Å). (C) Structure model of the predicted N-terminus of Amuc_1591. This model was then analyzed using DALI (2) to identify highly-similar PDB structures (table right). (D) Structure model of the predicted C-terminus of Amuc_1591. DALI analysis and functional predicts (table right). (E) Sequence independent alignment comparing the protein structures of Amuc_1591 (top) and L-fucokinase PDB: 9IIT (3). Structurally equivalent residues that are known to coordinate L-fucose within the active site of 9IIT are highlighted (arrows).

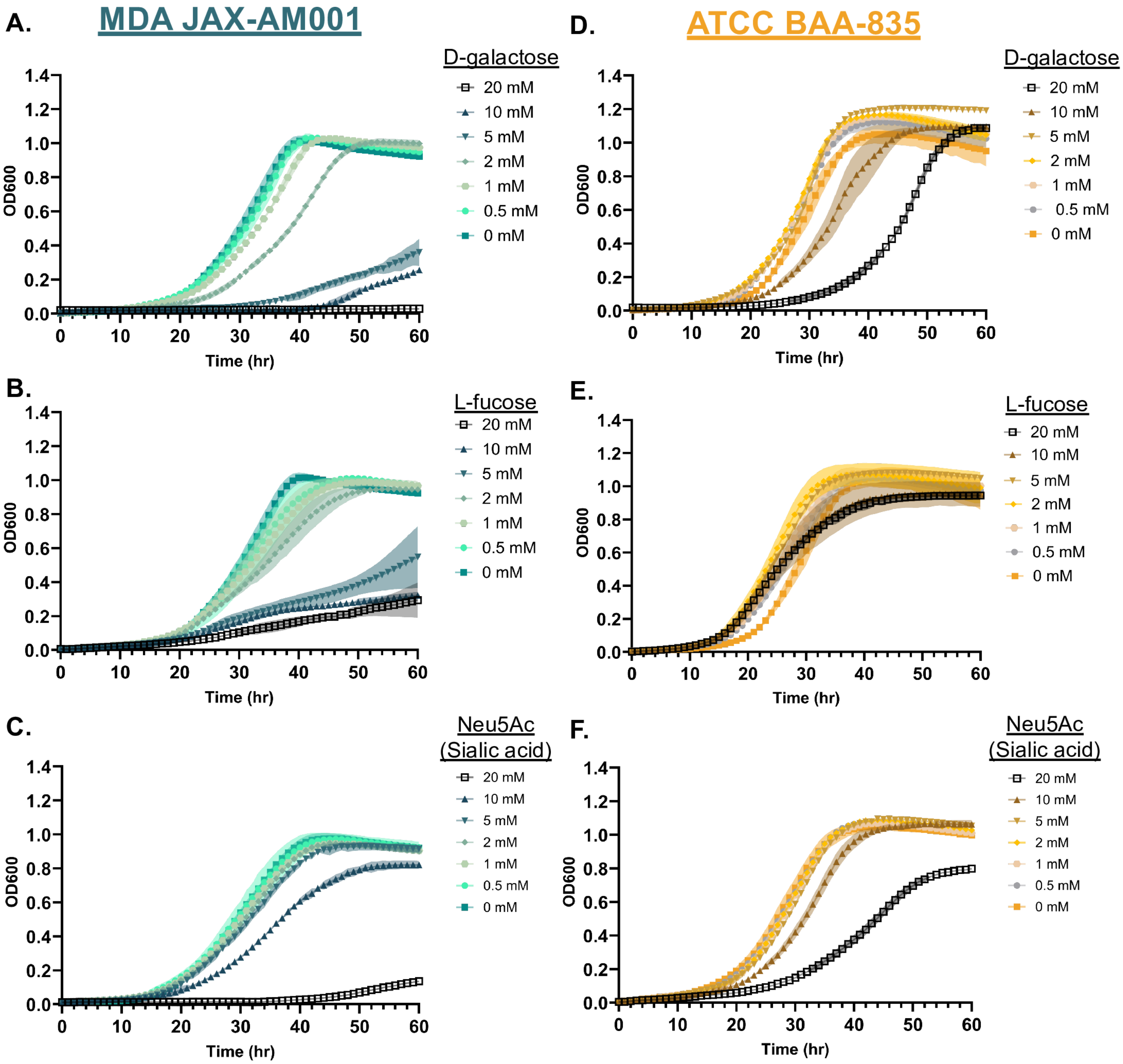

Supplemental Figure S6. Strain MDA JAX-AM001 is more sensitive to free glycan sugars than strain ATCC BAA-835. Growth data for strains MDA **(A-C)** and ATCC **(C-E)** grown in media supplemented with decreasing concentrations of D-galactose **(A, D)**, L-fucose **(B, E)**, and Neu5Ac **(C, F)**. Each growth condition was measured in triplicate for each strain.

A.

GalT Query (FASTA Sequences):
CAD6018840.1 (*E. coli*)
XMA43882.1 (*Clostridioides difficile* 630)
WP_093288623.1 (*Verrucomicrobium sp.* GASS474)
KIE58058.1 (*Methylacidiphilum kamchatkense*)

GalT Query (Domain search):

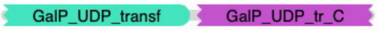

PF01087 / IPR005849 PF02744 / IPR005850

GalT Query (Structure homology):
E. coli GalT: AFDP A0A061YLT2

Method	Sequence similarity	Profile/HMM homology	Domain annotation	Structure homology
BLASTP (NCBI)	Ø (0 hits)	—	—	—
HMMER / jackhmmer	—	Ø (0 hits)	—	—
HHpred	Ø (0 hits)	Ø (0 hits)	Ø (0 hits)	—
InterProScan	—	—	Ø (0 domains)	—
Pfam-A (hmmscan)	—	Ø (0 hits)	Ø (0 domains)	—
Foldseek (PDB/AFDB)	—	—	—	Ø (0 matches)

Control protein	Sequence similarity	Profile/HMM	Domain annotation	Structure homology
GalK (galactose kinase: Amuc_0969)	✓	✓	✓	✓

B.

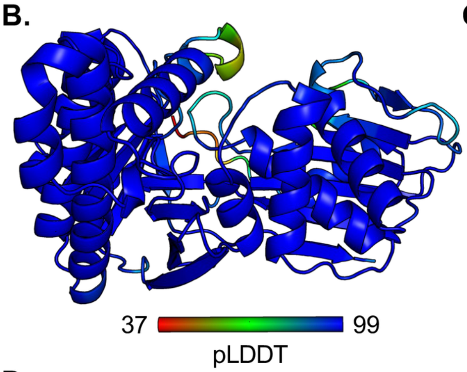

C.

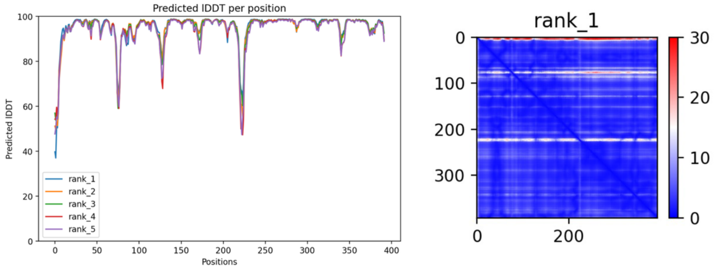

D.

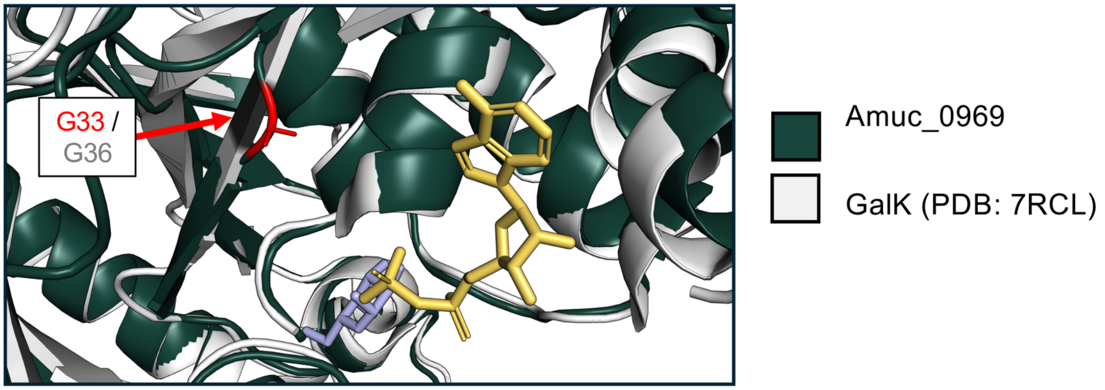

E.

```
Amuc_0969[ATCC]        -------MDLVQREISKETVTPYFIEYFGQAPTHVAAAPGRVNLIGEHTDYNNGFVMPMA   53
OOZ61_RS07480[MDA]     MGQNQSTMDLVQREISKETVTPYFIEYFGQAPTHVAAAPGRVNLIGEHTDYNNGFVMPMA   60
                              *****************************************************

Amuc_0969[ATCC]        LDNHCVVAVAPSPVGKHRFCGSLGDQIHEIAVEDALVPGEPFWSNYVRGVLANLHRRGIE   113
OOZ61_RS07480[MDA]     LDNHCVVAVAPSPVGKHRFCGSLGDQIHEIAVEDALVPGEPFWSNYVRGVLANLHRRGIE   120
                       ************************************************************

Amuc_0969[ATCC]        IGPVDMLIDSNVPRGGGLSSSAALEVAVCTALAAFAGVEIDPKEVALIGQAVEHEFVNVP   173
OOZ61_RS07480[MDA]     IGPVDMLIDSNVPRGGGLSSSAALEVAVCTALAAFAGVEIDPKEVALIGQAVEHEFVNVP   180
                       ************************************************************

Amuc_0969[ATCC]        CGIMDQFISANGKKGMALKLDCATLEYELVPMNNESVSVLVLDSAVKHSLADGAYGQRRK   233
OOZ61_RS07480[MDA]     CGIMDQFISANGKKGMALKLDCATLEYELVPMNNESVSVLVLDSAVKHSLADGAYGQRRK   240
                       ************************************************************

Amuc_0969[ATCC]        QCEEASSIMGVPSLREATLELLESFREQLGDVRYRRARHVIGENARVNAFANALARGDWD   293
OOZ61_RS07480[MDA]     QCEEASSIMGVPSLREATLELLESFREQLGDVRYRRARHVIGENARVNAFANALARGDWD   300
                       ************************************************************

Amuc_0969[ATCC]        EAGVAMRGSHASLRDDYEVSCAEVDTLVSLCDRIPSASSIYGARMTGGGFGGCIVALVKT   353
OOZ61_RS07480[MDA]     EAGVAMRGSHASLRDDYEVSCAEVDTLVSLCDRIPSASSIYGARMTGGGFGGCIVALVKT   360
                       ************************************************************

Amuc_0969[ATCC]        EDVEKVAQELLDGYCQETGIETTYLVTRAGEGARVLYQA 392
OOZ61_RS07480[MDA]     EDVEKVAQELLDGYCQETGIETTYLVTRAGEGARVLYQA 399
                       ***************************************
```

Supplemental Figure S7. Structure modeling of Amuc_0969 (GalK). (A) Representative GalT homologs from other bacteria including Verrucomicrobiales (thee order that includes *Akkermansia*, yellow bar) was queried against sequence databases using BLASTP, profile-based searches (HMMER/jackhmmer), domain annotation resources (InterProScan and Pfam), and structure-based similarity searching (Foldseek against PDB/AlphaFold DB) using default significance and coverage thresholds. No matches exceeded significance criteria in any search category. A positive-control protein (GalK; galactose kinase) recovered expected homologs/domains/structural matches using the same workflow (performed using GalK from *E. coli* NCBI Accession: CAD6018846.1; and PFAM domains: PF10509, PF00288, and PF08544). **(B)** Relaxed model of Amuc_0969 colored by pLDDT values (per residue measurement of local confidence, local distance difference test). **(C)** Plot of pLDDT score over the length of each of the 5 models that ColabFold generated (1). The highest ranked structural predict was used in this analysis. Position alignment error (PAE) plot for the highest ranked structure is shown (right). PAE is a measure of confident in the relative position of two residues within the predicted structure (colored by Å). **(D)** The active site of GalK (white, PDB: 7RCL (4)) bound with ATP (yellow) and galactose (blue). Alignment with Amuc_0969 (green) highlights the location of G33 (Amuc_0969) and the equivalent residue G36 (GalK) proximal to this active site. G33/G36 is located adjected to alpha-helix 3 (~5 Å). A G33R substitution is predicted to result in significant steric clashes. **(E)** Amino acid sequence alignment of each GalK homolog encoded by strains ATCC (top) and MDA (bottom).

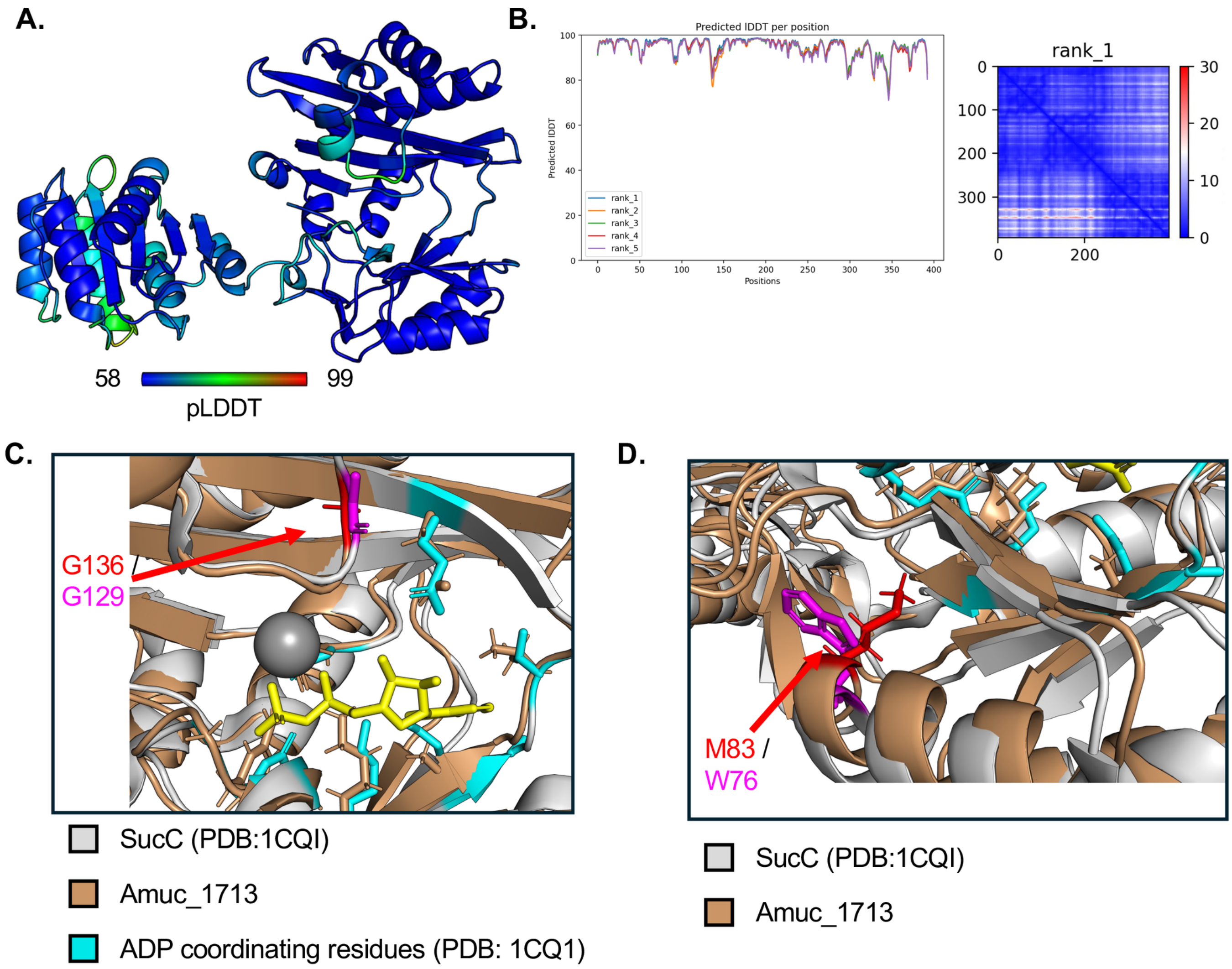

E.

```
Amuc_1713[ATCC]          -------MNIHEYQAKQLFERFGVATPKGIAASTAQEAAQTARNMGLSQYVVKAQVHAGG   53
OOZ61_RS00105[MDA]       MAKRPTVMNIHEYQAKQLFERFGVATPKGIAAATAQEAAQTARNMGLSQYVVKAQVHAGG   60
                                *************************:***************************

Amuc_1713[ATCC]          RGKGTFKNGFKGGVHVVKSVEEVEEVAGKMLNQVLVTKQTGEAGKLVSKIMVAEAVDLKK   113
OOZ61_RS00105[MDA]       RGKGTFKNGFKGGVHVVKSVEEVEEVAGKMLNQVLVTKQTGETGKLVSKIMVAEAVDLKK   120
                         ******************************************:*****************

Amuc_1713[ATCC]          ECYFAILQDRARECPVIVASTEGGMDIEEVAATRPEAIIREHINPALGILPFQALKIAVA   173
OOZ61_RS00105[MDA]       ECYFAILQDRARECPVIVASTEGGMDIEEVAATRPEAIIREHIDPALGILPFQALKIAVA   180
                         *******************************************:****************

Amuc_1713[ATCC]          LELTGPLLRQATKLITNVYKLFTALDCSLVEINPLVVTTDDRVCALDAKFNFDDNALYRH   233
OOZ61_RS00105[MDA]       LRLTGPLLRQATKLITNVYKLFTALDCSLVEINPLVVTTDDRVCALDAKFNFDDNALYRH   240
                         *.**********************************************************

Amuc_1713[ATCC]          PEIMEMRDETEEDPREVEAGKYDLNYIGLDGNIGCMVNGAGLAMATMDIIKYYGGEPANF   293
OOZ61_RS00105[MDA]       PEIMEMRDETEEDPREVEAGKYDLNYIGLDGNIGCMVNGAGLAMATMDIIKYYGGEPANF   300
                         ************************************************************

Amuc_1713[ATCC]          LDVGGSATEEMVTNAFRILTSDKNVKALLVNIFGGIMRCDVIAQGIVAAAKNIDMKIPLV   353
OOZ61_RS00105[MDA]       LDVGGSATEEMVTNAFRILTSDKNVKALLVNIFGGIMRCDVIAQGIVAAAKNIDMKIPLV   360
                         ************************************************************

Amuc_1713[ATCC]          VRLEGTNVEIGKKILADSGIAIIPADNLDEAAQKAVAAVK      393
OOZ61_RS00105[MDA]       VRLEGTNVEIGKKILADSGISIIPADNLDEAAQKAVAAVK      400
                         ********************:*******************
```

Supplemental Figure S8. Structure modeling of Amuc_1713 (SucC). (A) Relaxed model of Amuc_1713 colored by pLDDT values (per residue measurement of local confidence, local distance difference test). **(B)** Plot of pLDDT score over the length of each of the 5 models that ColabFold generated (1). The highest ranked structural predict was used in this analysis. Position alignment error (PAE) plot for the highest ranked structure is shown (right). PAE is a measure of confident in the relative position of two residues within the predicted structure (colored by Å). **(C)** The active site of SucC (white, PDB: 1CQI (5)) bound with ADP (yellow). Alignment with Amuc_1713 (green) highlights the equivalent residues G136 (Amuc_1713, red) and G129 (SucC, purple). The proximity to the active site and other coordinating residues suggest a G136S substitution affects ADP-binding. **(D)** Position of M83 (Amuc_1713, red) and equivalent residue W76 (SucC, purple) located near the active site. Substitution of M83K, a nonpolar residue with a polar residue at this position is predicted to destabilize β-sheets within the active site. **(E)** Amino acid sequence alignment of each SucC homolog encoded by strains ATCC (top) and MDA (bottom).

Table S1. Doubling times for strains ATCC BAA-835 and MDA JAX-AM001 grow in ADM supplemented with various sugars and sugar alcohols

Sugar (20 mM)	Strain	Doubling time (hr)	*p* - value	Strain	Doubling time (hr)	*p* - value
**ADM**	**BAA-835**	4.6 ± 0.1		**JAX-AM001**	5.4 ± 0.1	
**+NaCl**	**BAA-835**	4.1 ± 0.1	**	**JAX-AM001**	5.6 ± 0.2	ns
**+GalNAc**	**BAA-835**	3.9 ± 0.1	**	**JAX-AM001**	4.8 ± 0.2	*
**+Glucose**	**BAA-835**	4.0 ± 0.2	*	**JAX-AM001**	7.8 ± 0.5	**
**+Sucrose**	**BAA-835**	4.7 ± 0.6	ns	**JAX-AM001**	5.8 ± 0.5	ns
**+Fructose**	**BAA-835**	4.3 ± 0.2	*	**JAX-AM001**	6.7 ± 0.2	**
**+Xylose**	**BAA-835**	4.1 ± 0.1	*	**JAX-AM001**	5.2 ± 0.1	ns
**+Mannose**	**BAA-835**	3.9 ± 0.1	**	**JAX-AM001**	5.0 ± 0.2	*
**+Maltose**	**BAA-835**	4.4 ± 0.4	ns	**JAX-AM001**	5.4 ± 0.2	ns
**+Trehalose**	**BAA-835**	4.9 ± 0.5	ns	**JAX-AM001**	5.6 ± 0.3	ns
**+Sorbitol**	**BAA-835**	4.6 ± 0.2	ns	**JAX-AM001**	5.9 ± 0.5	ns
**+Mannitol**	**BAA-835**	4.8 ± 0.3	ns	**JAX-AM001**	5.8 ± 0.3	ns
**+Inositol**	**BAA-835**	4.6 ± 0.1	ns	**JAX-AM001**	5.7 ± 0.3	ns
**+Xylitol**	**BAA-835**	4.5 ± 0.3	ns	**JAX-AM001**	5.5 ± 0.1	ns
**+Arabitol**	**BAA-835**	4.6 ± 0.6	ns	**JAX-AM001**	7.2 ± 0.2	**
**+Galactose**	**BAA-835**	6.0 ± 0.4	*	***JAX-AM001***	*No growth*	--
**+Lactose**	**BAA-835**	3.9 ± 0.1	**	**JAX-AM001**	8.9 ± 0.3	**
**+L-Fucose**	**BAA-835**	4.9 ± 0.3	ns	**JAX-AM001**	7.9 ± 0.3	**
**+D-Ribose**	**BAA-835**	4.5 ± 0.2	ns	**JAX-AM001**	6.5 ± 0.4	*
**+L-Arabinose**	**BAA-835**	4.7 ± 0.1	ns	**JAX-AM001**	5.2 ± 0.2	ns
+D-Arabinose	***BAA-835***	*No growth*	--	***JAX-AM001***	*No growth*	--
**+Sialic Acid**	**BAA-835**	7.4 ± 0.20	**	**JAX-AM001**	7.9 ± 0.5	**

*** < 0.01**

**** < 0.001**

Dataset S1. Breseq SNP calls and the genomic position of D-arabinose suppressor mutations within the genome of strains ATCC BAA-835 and JAX-AM001.

Dataset S2. Breseq SNP calls and the genomic position of D-galactose suppressor mutations within the genome of strain JAX-AM001.

Dataset S3. Breseq SNP calls and the genomic position of L-fucose suppressor mutations within the genome of strain JAX-AM001.

Dataset S4. Primers used in this study to confirm mutations.

Dataset S5. Protein sequences, gene name, and NCBI accession number for each protein that was used to generate protein structure models.

SI References

1. M. Mirdita, *et al.*, ColabFold: making protein folding accessible to all. *Nat Methods* **19**, 679–682 (2022).
2. L. Holm, A. Laiho, P. Törönen, M. Salgado, DALI shines a light on remote homologs: One hundred discoveries. *Protein Science* **32**, e4519 (2023).
3. S.-W. Lin, *et al.*, Structural insight into the catalytic mechanism of the bifunctional enzyme l-fucokinase/GDP-fucose pyrophosphorylase. *Journal of Biological Chemistry* **301**, 108344 (2025).
4. L. Liu, *et al.*, Structure-Based Optimization of Small Molecule Human Galactokinase Inhibitors. *J. Med. Chem.* **64**, 13551–13571 (2021).
5. M. A. Joyce, M. E. Fraser, M. N. G. James, W. A. Bridger, W. T. Wolodko, ADP-Binding Site of Escherichia coli Succinyl-CoA Synthetase Revealed by X-ray Crystallography,. *Biochemistry* **39**, 17–25 (2000).
